# Unravelling Multilayered RNA Modification Networks in Female Reproduction and Obstetric/Gynaecologic Disorders

**DOI:** 10.3390/biom16040571

**Published:** 2026-04-13

**Authors:** Yujie Kuai, Yanjun Yi, Xinyu Li, Zhuangping Wang, Yan Zheng, Yuxuan Li, Yulin Li

**Affiliations:** 1 The Main Campus, Chengdu University of Traditional Chinese Medicine, Chengdu 611137, China; 2 School of Medical and Life Sciences, Chengdu University of Traditional Chinese Medicine, Chengdu 611137, China; 3 School of Clinical Medicine, Chengdu University of Traditional Chinese Medicine, Chengdu 611137, China

**Keywords:** RNA modification, female reproduction, obstetrical, gynaecological disease, reproductive physiology, gynaecological oncology, pregnancy-related diseases, reproductive endocrine diseases

## Abstract

**Background/Objective:** RNA modifications, including N^6^-methyladenosine (m^6^A), 5-methylcytosine (m^5^C), 7-methylguanosine (m^7^G), N^1^-methyladenosine (m^1^A), pseudouridine (Ψ), N^4^-acetylcytidine (ac^4^C), 5-methoxycarbonylmethyl-2-thiouridine (mcm^5^s^2^U) and adenosine-to-inosine (A-to-I) editing, constitute a critical layer of post-transcriptional regulation that influences RNA stability, splicing, translation and degradation. This review aims to systematically summarise the current understanding of the molecular mechanisms and regulatory networks of RNA modifications in the female reproductive physiology and to evaluate their pathological implications in obstetric and gynaecologic disorders. **Methods:** We conducted a comprehensive literature review, synthesising findings from high-throughput sequencing studies, functional experiments and clinical investigations. The review integrates evidence across multiple RNA modification types, their regulatory enzymes (writers, erasers and readers) and their roles in physiological processes (germ cell development, oocyte maturation, embryogenesis and endometrial function) and pathological conditions (gynaecologic cancers, preeclampsia, endometriosis, polycystic ovary syndrome and premature ovarian insufficiency). **Results:** RNA modifications function as dynamic and reversible regulators that orchestrate key reproductive events, including primordial germ cell differentiation, oocyte meiosis, the maternal-to-zygotic transition, the establishment of uterine receptivity, and placental development. These modifications operate through coordinated writer–eraser–reader networks that fine tune transcripts’ stability, translation efficiency and RNA decay. The dysregulation of these epitranscriptomic networks is strongly implicated in the pathogenesis of gynaecologic malignancies (cervical, ovarian, endometrial cancers and choriocarcinoma), pregnancy-related disorders (preeclampsia, gestational diabetes mellitus and recurrent miscarriage), reproductive endocrine disorders (polycystic ovary syndrome and premature ovarian insufficiency) and benign gynaecological conditions (endometriosis and adenomyosis). Emerging evidence also reveals complex crosstalk among RNA modifications, such as cooperative interactions between m^6^A and m^5^C in translation regulation and antagonistic relationships between m^6^A and A-to-I editing. **Conclusions:** RNA modifications represent an essential and multifaceted regulatory layer in female reproduction, with broad implications for disease pathogenesis. Their unique reversibility and context-dependent functions offer promising opportunities for the development of diagnostic biomarkers and targeted therapeutic interventions. Future researchers should prioritise integrated multi-omics approaches, enhanced human-relevant models and clinical translation to fully realise the potential of epitranscriptomic medicine in reproductive health.

## 1. Introduction

The proper functioning of female reproductive health depends on the proper functioning of oogenesis, embryogenesis and gestation [1]. The imbalance of the body’s natural rhythm can lead to a variety of disorders, including infertility and gynaecological cancer [2]. These conditions impose a serious burden on global health. In 2022, an estimated 660,000 new cases of cervical cancer occurred, resulting in an estimated 350,000 deaths. Five-year survival ratios for advanced (metastatic) stages of cervical cancer remain under 20% [3,4]. According to the World Health Organization (WHO), without effective intervention, global cervical cancer deaths are projected to increase to approximately 460,000 annually by 2040, with low- and middle-income countries facing the greatest relative increase [5]. Malignancies of the ovary present no less serious challenges, with the WHO forecasting a 42% rise in global incidence by 2040, driven largely by ageing populations and the lack of effective early detection strategies. The prognosis remains less than 50% in most patients despite aggressive therapeutic intervention [6,7]. Beyond neoplastic conditions, polycystic ovary syndrome (PCOS) represents another significant gynaecologic disorder, affecting 4–21% of women. This condition compromises fertility and predisposes individuals to metabolic disturbances. Its prevalence is expected to rise globally in parallel with increasing rates of obesity and metabolic syndrome [8]. The molecular basis governing these diseases must be understood to develop novel diagnostic and therapeutic strategies because of clinical realities.

Moreover, RNA modifications are attracting increasing attention in the epigenetics field, as they make up the primary regulatory layer of the epitranscriptome, which provides a dynamic, information-rich layer for gene regulation above and beyond DNA sequence information [9,10]. Modifications that altered the chemical properties and three-dimensional shapes of the RNA molecule and the molecule’s ability to interact with proteins in a specific manner controlled the splicing, stability, nucleocytoplasmic transport, translation efficiency and degradation of RNA [11,12]. Modifications that altered the chemical properties and three-dimensional shapes of the RNA molecule, as well as the ability of the molecule to participate in protein interaction, in a specific manner controlled the splicing, stability, nucleocytoplasmic transport, translation efficiency and degradation of RNA [13,14] (Table 1). The DNA methylation and the chromatin remodelling that accompany histone modification are relatively stable. However, some reversible RNA modifications, such as m^6^A, show faster dynamics and environmental responsiveness. The modifications may accomplish the reprogramming of particular transcripts within minutes or hours of stimulation. This feature makes RNA modification a highly effective regulatory mechanism in the quick adaptation of cells to physiological variations and stress responses [15,16].

According to studies, females’ physiological and pathological processes, ranging from reproductive health and embryonic development to cancer development, are influenced by epigenetic regulation [61,62,63]. Although meaningful advances have been made, many knowledge gaps remain, and translating these advances into the clinic is highly challenging [64,65]. Review articles often concentrate on certain forms of m^6^A regulation, such as tumour m^6^A or the role of m^6^A in fertility and pregnancy [66,67]. Few integrated studies connect female reproductive biology and its associated pathologies while addressing RNA modifications beyond m^6^A. In addition, the activity of m^6^A-modifying enzymes greatly depends on the cell type. Methyltransferase-like 3 (METTL3) is reportedly a tumour promoter in ovarian cancer cells [68]. However, it performs an anti-tumour function in the host myeloid immune cell by inhibiting the recruitment of myeloid-derived suppressor cells (MDSCs) [69]. This dual function acts in an opposite direction and can be responsible for epigenetic regulation, which is context dependent and complicates translation efforts.

In this article, the researchers summarise the basic concepts and mechanisms of m^6^A and other RNA modifications such as m^5^C, m1A and A-to-I. Likewise, they summarise the pathological evidence across normal female reproductive processes, benign disorders, including infertility-associated conditions and gynaecological cancers. We also discuss the diagnostic and therapeutic value of relevant biomarkers and note both the possibilities and the limitations in the field. We also looked at recent changes in detection technologies for RNA modifications. Finally, we suggest future research directions to improve the current understanding of the epitranscriptome in female health and to guide the development of novel therapeutic interventions.

## 2. RNA Modification

**Figure 1:**
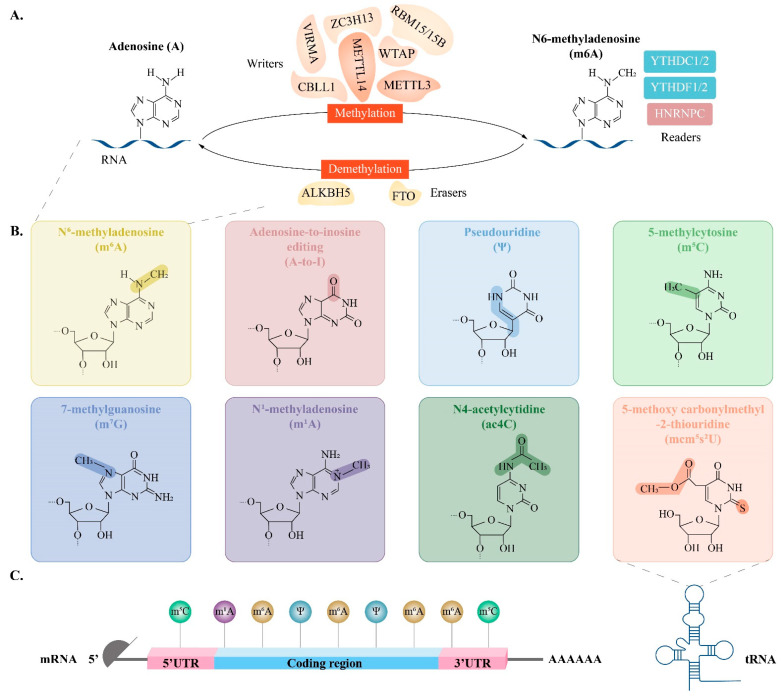
Molecular mechanisms of RNA modifications and their distribution on mRNA and tRNA. (**A**). Dynamic regulation of RNA N6-methyladenosine (m6A) modification. The m6A landscape is coordinately regulated by “Writers” (methyltransferase complex consisting of METTL3, METTL14, WTAP, RBM15/15B, ZC3H13, VIRMA, and CBLL1), “Erasers” (demethylases FTO and ALKBH5), and “Readers” (binding proteins such as YTHDC1/2, YTHDF1/2, and HNRNPC) that execute specific biological functions. (**B**). Chemical structures of diverse RNA modifications. Schematic representation of representative RNA modifications, including N6-methyladenosine (m6A), Adenosine-to-Inosine (A-to-I) editing, Pseudouridine (Ψ), 5-methylcytosine (m5C), 7-methylguanosine (m7G), N1-methyladenosine (m1A), N4-acetylcytidine (ac4C), and 5-methoxycarbonylmethyl-2-thiouridine (mcm5s2U). Modified chemical groups are highlighted. (**C**). Distribution patterns of RNA modifications. Various modifications are distributed across the 5′ untranslated region (5′UTR), coding region (CDS), and 3′ untranslated region (3′UTR) of mRNA, as well as specific sites on the cloverleaf structure of tRNA.

RNA modifications are covalent chemical changes in RNA molecules that regulate RNA molecules’ metabolism and function by changing their chemical structure. The most abundant and well-characterised modifications include m^6^A, m^5^C and Ψ, which tightly control gene expression and play an important role in biological processes such as immune cell regulation, tumorigenesis and neurological disorders [70] (Figure 1).

### 2.1. M^6^a Modification

#### 2.1.1. Molecular Basis and Regulatory Mechanisms

The N6-methyladenosine (m^6^A) modification represents the most abundant and dynamically reversible internal chemical modification within eukaryotic mRNA. The homeostasis of this modification relies on the synergistic regulation of three classes of functional proteins known as methyltransferases (Writers), which catalyse installation; demethylases (Erasers), which perform removal and m^6^A binding proteins (Readers), which are responsible for recognition. These proteins achieve post-transcriptional regulation through the addition, removal and decoding of a modification [71,72].

Writers are responsible for catalysing the installation of m^6^A, and their core members include the Methyltransferase family (METTL3/14/16) and Wilms Tumour 1-Associated Protein (WTAP) [73]. Conversely, demethylases act as Erasers, such as the Alpha-ketoglutarate-dependent dioxygenase family (ALKBH9/5/3/1), and these enzymes can remove the m^6^A modification to reverse the methylation state [74]. Readers are recognition proteins, including YTH domain family proteins (YTHDF1/2/3 and YTHDC1/2), insulin-like growth factor 2 mRNA-binding proteins (IGF2BPs) and heterogeneous nuclear ribonucleoproteins (hnRNPs). It communicates downstream biological effects by recognised binding to the m^6^A site [75].

To address the dynamic and context-dependent nature of m6A regulation, it is important to distinguish that different writer–reader axes preferentially regulate distinct RNA metabolic processes. For instance, METTL3–YTHDF1 primarily enhances translation efficiency, whereas METTL3–YTHDF2 mainly promotes mRNA decay, and the METTL3–IGF2BP axis stabilises transcripts. This functional divergence explains why similar METTL3-mediated m6A events can lead to different biological outcomes across cellular contexts [76].

The dynamic reversibility of this regulatory network guarantees that m^6^A modifications can respond to various intracellular and extracellular signals. Correspondingly, m^6^A is implicated in nearly every stage of RNA metabolism, including RNA splicing, nuclear export and the regulation of stability and translation efficiency [77].

M^6^A modification follows a sequential regulatory logic: methylation by METTL3/METTL14/WTAP, complex, dynamic removal by FTO/ALKBH5 and functional decoding by reader proteins, which direct RNA towards translation (YTHDF1), degradation (YTHDF2) or stabilisation (IGF2BPs). This writer–eraser–reader axis forms the core framework of m6A-mediated gene regulation [28,78].

#### 2.1.2. M^6^a Related Non-Coding RNAs

Apart from RNA, which codes for proteins, m^6^A modification occurs frequently among various classes of ncRNA. These transcripts, which comprise approximately 98% of the total human transcriptional output, were previously thought to be junk RNA and are now considered key regulators of gene expression [79]. Long non-coding RNAs (lncRNA), circular RNA (circRNA), microRNA (miRNA) and PIWI-interacting RNA (piRNA) are among the classes that do not code proteins. Through interconnected RNA interaction networks, they take part in germ cell development, embryogenesis and the progression of gynaecologic malignancies [80,81,82].

M^6^A modification occurs not only on mRNA but also on diverse ncRNA molecules. By shaping stability, translation and decay, m^6^A helps coordinate processes, such as oocyte maturation, embryo endometrium communication and gynaecologic cancer progression [13,83].

Long non-coding RNAs (lncRNAs) are non-coding transcripts longer than 200 nucleotides that constitute roughly 80% of ncRNA. Depending on their function, they can work as signals, decoys, guides or scaffolds [80]. In cervical cancer, METTL3-mediated m^6^A modification stabilises lncRNA FOXD2 AS1, which recruits LSD1 to the p21 promoter to suppress p21 expression and, thus, promote tumour cell proliferation and migration [84]. In ovarian cancer, METTL3-mediated m^6^A modification stabilises lncRNA LINC00857, activates YAP TEAD signalling and enhances stem-like features and metastatic potential [85]. Notably, these examples represent a ‘stability-enhancing’ m6A mechanism primarily mediated through IGF2BP recognition, distinguishing them from the miRNA-related processing mechanisms described below. ALKBH5-mediated m^6^A demethylation stabilises lncRNA PVT1 and promotes tumour cell proliferation, invasion and chemotherapy resistance, at least partly through increasing FOXM1 expression.

Additionally, circRNAs are covalently closed RNA molecules generated by back splicing. They are highly stable and can influence tumours’ behaviour by sequestering miRNAs or interacting with proteins [86]. In cervical cancer, circARHGAP12 interacts with IGF2BP2 through its m^6^A-modified sites, enhances FOXM1 mRNA stability and supports the formation of a circARHGAP12 IGF2BP2 FOXM1 complex that promotes cell proliferation and migration [87]. In endometrial cancer, m^6^A-modified circCHD7 also binds IGF2BP2 and increases PDGFRB mRNA stability in an m^6^A-dependent manner, which activates JAK/STAT signalling and drives tumours’ growth and progression [88]. These findings highlight a consistent m6A–IGF2BP–mRNA stabilisation axis in circRNA-mediated oncogenic pathways, which are mechanistically distinct from miRNA maturation pathways. ALKBH5-mediated demethylation reduces circCCDC134 stability, affecting metastasis through miRNA sequestration and transcriptional regulation [89].

MiRNAs are the endogenous single-stranded non-coding RNAs of about 22 nucleotides. They primarily bind the three prime untranslated regions of target mRNAs to inhibit translation or promote degradation. They also shape processes such as the cell cycle, proliferation and apoptosis [90]. In cervical cancer, METTL3-mediated m^6^A modification reduces the expression of the tumour-suppressive miR 193b, leading to the overexpression of its target CCND1 and promoting invasion and deep stromal infiltration [91]. Conversely, miR 30c 5p suppresses METTL3, reduces m^6^A modification on KRAS mRNA, induces ferroptosis in cervical cancer cells and inhibits tumours’ growth and metastasis [92]. In choriocarcinoma, METTL3 promotes the maturation of pri miR 935 in an m^6^A-dependent manner. The resulting increase in miR 935 enhances proliferation, migration, invasion and angiogenesis by targeting Connexin 43 (Cx43), showing a pro-tumour m^6^A miRNA axis in gestational trophoblastic disease [93]. Unlike lncRNAs and circRNAs, m6A modification in miRNAs primarily regulates pri-miRNA processing through DGCR8 recognition, representing a distinct RNA maturation control mechanism rather than stability regulation.

PIWI-interacting RNA (piRNAs) are small non-coding RNAs that bind PIWI proteins and can exert both tumour-promoting and tumour-suppressive effects in cancer. In cervical cancer, piRNA 14633 increases the stability and expression of METTL14 mRNA in a concentration-dependent manner, elevates CYP1B1 expression and promotes cell proliferation, migration and invasion [94]. In addition, piRNA 17458 enhances WTAP mRNA stability without altering METTL3 14, ALKBH5 or FTO, increases global m^6^A levels and supports tumorigenesis [95]. However, piR 26441 is expressed at low levels in ovarian cancer and demonstrates tumour suppression. It interacts with YTHDC1 and prevents the tripartite motif, containing 56 (TRIM56)-mediated ubiquitin-dependent degradation, thus stabilising the YTHDC1 protein. This effect promotes the m^6^A-dependent decay of the mitochondrial translation elongation factor TSFM mRNA, reduces oxidative phosphorylation, heightens reactive oxygen species, triggers DNA damage-related apoptosis and finally restrains malignant proliferation and tumour formation in vivo [96].

M^6^A regulators (METTL3/WTAP/ALKBH5) modify ncRNAs (lncRNA, circRNA, miRNA and piRNA), which subsequently regulate downstream oncogenic pathways through three main routes: stabilising oncogenic transcripts (e.g., FOXM1 and PDGFRB), modulating miRNA maturation and target repression (e.g., CCND1 and KRAS) and reshaping signalling pathways such as PI3K–AKT, JAK–STAT and YAP–TEAD. These interconnected networks ultimately control proliferation, metastasis, metabolism and therapy resistance in gynaecologic cancers [97].

### 2.2. Adenosine to Inosine RNA Editing

Apart from m^6^A modification, adenosine-to-inosine (A-to-I) RNA editing is one of the most common post-transcriptional RNA modifications in mammals [98]. This process is performed by the adenosine deaminase acting on the RNA family that acts on double-stranded RNA, including the catalytically active adenosine deaminase acting on RNA1 (ADAR1), ADAR2 and ADAR3, which usually lacks catalytic activity [99]. Biochemically, ADAR enzymes convert adenosine in RNA into inosine through hydrolytic deamination [100]. Because inosine resembles guanosine in its structure, the translation machinery often interprets it as guanosine, which can introduce nonsynonymous changes in protein coding sequences [101]. Besides changing the amino acid’s identity and expanding proteomic diversity, A-to-I editing can affect RNA splicing, secondary structures, transcript stability and translational efficiency [102]. Editing from A to I is another common occurrence in ncRNAs and may interfere with either Drosha or Dicer processing. This positively affects miRNA maturation and target recognition while negatively influencing circRNA biogenesis [103].

Beyond m^6^A, another prevalent and functionally significant RNA modification is A-to-I editing. A-to-I editing plays a central role in the female reproductive system’s functioning and maladies. In oocytes, inosine marks are enriched in coding regions and are preferentially found at wobble sites, which suggests that A-to-I editing may alter codon usage and affect the clearance and stability of maternal RNA in meiosis [104]. Ovarian granulosa cell functioning also requires ADAR1-mediated editing, as the granulosa cell-specific deletion of ADAR1 impairs ovulation, delays oocyte maturation and disrupts the expression of inflammation-related genes [105]. When A-to-I editing is unregulated, it can promote the development of POCS [106]. Patients with polycystic ovary syndrome experience increasing RNA editing sites in adipose tissue and the abnormally reduced expression of ADAR2 in granulosa cells [107]. ADAR1 influences the RNA editing of substrates, such as Eukaryotic Translation Initiation Factor 2-Alpha Kinase 2 (EIF2AK2), which increases expression and activates Mitogen-Activated Protein Kinase (MAPK) signalling. Hence, the disease-associated pathology is worsened. Thus, this site may have good potential for clinical diagnoses [108].

A-to-I editing also shapes the immune microenvironment outside its direct functioning in the regulation of reproductive endocrines. ADAR1 can edit endogenous double-stranded RNA, including Alu-derived transcripts, converting them to less immunogenic versions. This prevents them from being recognised by innate immune sensors, such as melanoma differentiation-associated protein 5, thus diminishing self-directed inflammation and immune activation [32]. A-to-I editing also interacts with other RNA modifications such as m^6^A. Together, these form an integrated post-transcriptional regulatory network [109]. Selectively targeting ADAR enzymes is becoming a viable research approach because of its importance to oocyte development and immune homeostasis [110]. Inhibitors of ADAR1 have been created, and these epitranscriptomic therapies may provide novel biomarkers and precision treatments for polycystic ovary syndrome and numerous female reproduction-related diseases [111].

### 2.3. Pseudouridine

One of the most prevalent RNA modifications found in eukaryotes is pseudouridine (Ψ), termed RNA’s fifth nucleotide when first identified in the 1950s. The Ψ isomer of uridine is a C-glycoside. The conversion of uridine to Ψ affects ribose conformation and increases the backbone’s rigidity, thus stabilising RNA duplex structures [112]. There are two major routes to pseudouridylation. One modification route uses standalone Ψ syntheses that directly encounter the substrate and catalyse the chemical transformation. The other pathway relies on the box H ACA ribonucleoprotein complex containing the Dyskerin (DKC1) protein, which facilitates site-specific catalysis [113]. The modification appears irreversible because the carbon–carbon bond formed between the base and ribose by Ψ is more chemically inert and more difficult to break than the carbon–nitrogen bond in uridine. In fact, there is no identified eraser, and only a few reader proteins have been reported, such as Profilin 1 (PFN1), polyadenylate binding protein 1 (PABPC1) and the yeast factor Prp5 [38].

Additionally, Ψ is in different areas of mRNA and regulates many post-transcriptional processes, namely splicing, translation and transcript stability. While being translated, codons with Ψ can encourage aberrant base pairing. This can cause amino acid misincorporation or stop codon readthrough. Moreover, Ψ protects RNA from degradation by ribonucleases. This increases the half-life of transcripts [112,113]. Changes in environmental signals and cellular stress can alter Ψ landscapes. Disruptions to these programmes occur via mutations in Ψ synthases or through their aberrant expression, leading to a range of human diseases [113].

An alteration of Ψ has significant clinical applications in female reproductive disorders and gynaecologic cancers. Because human cells lack the requisite enzymes to metabolise C-glycoside compounds efficiently, excess Ψ is excreted and may accumulate in many human body fluids [113]. Elevated Ψ has been detected in plasma samples collected before a diagnosis of ovarian cancer, indicating a relationship between dysregulated Ψ and preclinical disease progression that may present an early warning biomarker [114]. A candidate prognostic marker and therapeutic target in ovarian cancer, Ψ synthase 7 (PUS7) promotes tumour cell proliferation [115]. The enrichment of DKC1 protein levels is also related to breast cancer progression via effects on RNA biogenesis and telomerase activity regulation [116]. The findings enhance our understanding of how Ψ modification functions at the cellular level, provide an alternative view of disease evolution within the female reproductive system and bolster prospects for personalised clinical applications.

### 2.4. 5-Methylcytidine

At the fifth carbon of cytosine, 5-Methylcytidine (m^5^C) is a post-transcriptionally regulated RNA chemical modification. This modification was again found in tRNA, rRNA and mRNA and plays an important role in epitranscriptomic regulation [117]. The installation of m^5^C requires coordination between various protein complexes. Major methyltransferases include members of the NSUN family and DNA methyltransferase 2 (DNMT2), otherwise called tRNA aspartic acid methyltransferase 1 (TRDMT1). The NSUN family has seven members, NSUN1 to NSUN7, and is named after the highly conserved NOL1/NOP2/SUN domain. The core enzymes involved in the mRNA m^5^C modification include NSUN2 and NSUN6 [118]. This modification is reversible because ten-eleven translocation (TET) and AlkB homologue 1 (ALKBH1) can oxidise m^5^C back to 5-hydroxymethylcytidine, reversing the methylation event [119]. Functionally, m^5^C mainly works through recognition by reader proteins that determine RNAs’ fate. As an example, the nuclear reader Aly/REF export factor (ALYREF) acts as an mRNA nuclear export factor by recognising m^5^C sites, whereas the cytoplasmic reader Y-box-binding protein 1 (YBX1) recruits cofactors to enhance mRNAs’ stability and translation. These mechanisms display how essential m^5^C is to maintaining the balance of the RNA metabolism [45,120].

The m^5^C modification in female reproductive physiology and pathology is spatio–temporally regulated. The levels of m^5^C vary in oocytes and early embryos. NSUN5-mediated rRNA methylation plays a crucial role in the stability of maternal mRNA and when the loss function NSUN5 can cause ovarian insufficiency and developmental arrest [121]. During the endometrial cycle, epithelial cytoskeleton remodelling and adhesion molecule expression regulated by NSUN2-mediated m^5^C modification are needed to establish normal endometrial receptivity and embryo implantation [122]. Abnormal m^5^C modification is associated with different gynaecologic diseases. Altered TRDMT1 activity in granulosa cells can disrupt DNA damage repair mechanisms and lead to premature ovarian insufficiency [123]. In cancers such as cervical cancer, the high expression of NSUN2 causes aberrant methylation of the oncogenic mRNAs Keratin 13 (KRT13) and Leucine-rich repeat-containing eight volume-regulated anion channel A (LRRC8A). These methylated transcripts are recognised and stabilised by the reader protein YBX1, enhancing tumour cell proliferation, invasion and chemotherapy resistance. These findings suggest that the m^5^C pathway may serve as a novel biomarker and therapeutic target for gynaecologic diseases [124,125].

### 2.5. 7-Methylguanosine

N7-Methylguanosine (m7G) is a common epitranscriptomic modification in RNA. It involves methylation at the N7 position of guanosine catalysed by specific methyltransferases [126]. In addition, m7G appears mainly at the 5 prime cap and internal sites of eukaryotic mRNA and is also found in the internal positions of rRNA, tRNA and miRNA [127]. Key m7G regulatory proteins include complexes formed by methyltransferase-like 1 (METTL1) with WD repeat domain 4 (WDR4), Williams–Beuren syndrome chromosome region 22 (WBSCR22) with tRNA methyltransferase activator subunit 11-2 (TRMT112) and RNA guanine-7 methyltransferase (RNMT) with its activating mini-protein (RAM) [128]. The METTL1 WDR4 heterodimer installs m7G at the tRNA variable loop G46, G quadruplex structures of primary microRNAs (pri-miRNAs) and internal mRNA sites, regulating RNA stability, nuclear export and translation efficiency [129]. WBSCR22 and TRMT112 work together to catalyse m7G at G1639 of 18S rRNA, supporting ribosome biogenesis and maturation [130]. RAM and RNMT function to create an m7G cap at the 5′ end of the mRNA. Furthermore, its interaction with eukaryotic translation initiation factor 4E (eIF4E) directly influences nuclear export, and translation initiation states this assertion [131]. Outside these roles, m7G and N6,2 prime-O-dimethyladenosine (m^6^Am) stave off the degradation mediated by mRNA-decapping enzyme 2 (DCP2), thus maintaining the stability of RNA [132].

The m7G alteration and dysregulation of regulatory protein are partially implicated in the development of tumours and remodelling of the immune microenvironment in female reproductive malignancies [133]. M7G-associated lncRNAs and miRNAs in endometrial cancer may be used to build prognostic models, with expression levels significantly correlated with immune infiltration profiles and drug sensitivity [134]. Evidence from breast cancer research has suggested that m7G modification facilitates the infiltration of multiple immune cells. Examples include naïve B cells, CD4 plus memory T cells, CD8 plus T cells, NK cells and M1 macrophages. Most importantly, nuclear cap-binding protein 1 (NCBP1) mRNA is identified as a key m7G target [135]. The m7G tRNA modification mediated by METTL1 can affect the translation of chemokines such as CXCL8, which will recruit polymorphonuclear myeloid-derived suppressor cells (PMN-MDSCs) to create an immunosuppressive tumour microenvironment [136]. In ovarian cancer, prognostic models based on m7G regulatory genes can effectively predict patients’ survival outcomes [137]. The m7G modification, along with m^6^A and m^5^C modifications on RNA forming multi-layered networks, underlies the escape ability of the immune system and therapy resistance in gynaecologic malignancies [138]. The precise function of m7G in shaping a tumour’s immune microenvironment indicates its potential as a biomarker and a therapeutic target. Monitoring its modification status may help guide precision immunotherapy [139].

### 2.6. N1-Methyladenosine

N1 methyladenosine (m^1^A) refers to the methylation modification that occurs at the first nitrogen of adenosine. This modification takes place in both coding and non-coding RNAs [140]. M^1^A carries explain a positive charge under physiological conditions, which disrupts standard Watson–Crick base pairing, alters the secondary structure of RNA and affects RNA–protein interactions. M^1^A is involved in controlling RNA stability and translation efficiency, as well as other internal metabolism processes [141]. Methyltransferases, demethylases and reader proteins stringently control the balance of m^1^A. Key methyltransferases are tRNA methyltransferase 6 (TRMT6), TRMT61A, TRMT10C, TRMT61B and nucleomethylin (NML). Demethylases that erase m^1^A include AlkB homologue 1 (ALKBH1), ALKBH3, ALKBH7 and fat-mass and obesity-associated protein (FTO). Recognising proteins include YTH domain-containing proteins YTHDF1, YTHDF2, YTHDF3 and YTHDC1 [142].

In the female reproductive system and gynaecologic diseases, m^1^A modification and its regulatory factors reveal clear clinical and pathological importance [143]. In ovarian and breast cancers, the demethylase ALKBH3 removes m^1^A modifications from GC-rich regions of colony stimulating factor 1 (CSF1) mRNA, markedly prolonging its half-life and promoting macrophage recruitment and cancer cell invasion within a tumour’s microenvironment [144]. High expression of the methyltransferase TRMT6 in ovarian cancer is tied to poor patient survival, while microRNA 1915p can target TRMT6 to reduce its oncogenic effects [145]. In cervical cancer, the methyltransferase TRMT10C works as an oncogene with elevated expression tied to worse survival, whereas TRMT6, TRMT61A, ALKBH3, YTHDC1 and YTHDF2 display lower expression and act as tumour suppressors [146]. In addition, in endometrial cancer, m^1^A-related lncRNAs can be used to build prognostic models tied closely to immune infiltration patterns and drug sensitivity [147]. In summary, m^1^A modification adjusts metabolic networks and immune infiltration in gynaecologic malignancies and is becoming a target for developing new therapeutics and combinatory immunotherapies [148].

### 2.7. N4-Acetylcytidine

N4-acetylcytidine (ac4C) is an RNA epitranscriptomic modification. The modification was identified in the tRNA of yeast in 1966 and was confirmed in the tRNA of bacteria in 1972 and in 18S rRNA of eukaryotes 1978 [149,150,151]. As the sole known RNA modification in eukaryotes, ac4C is produced by N-acetyltransferase 10 (NAT10), which has acetyltransferase, tRNA binding and RNA helicase domains. Studies have demonstrated that ac4C exists in different types of RNAs, including viral RNAs [152,153]. It assists with the assembly of RNA duplex and modulates translation. The ac4C modification in the anticodon loop of tRNA enhances codon recognition, while ac4C modification in the decoding centre of 18S rRNA helps the ribosome to select the correct codons. Transcript stability is enhanced due to ac4C modifications of mRNA coding regions. These mechanisms maintain and enhance, respectively, the fidelity and efficiency of target protein translation [154,155].

Regarding female reproductive health, NAT10-mediated ac4C modification is associated with oogenesis, embryonic development and various gynaecological diseases [153]. NAT10 expression slowly drops between the germinal vesicle stage and metaphase II (MII) during oocyte maturation, and this event influences meiotic progression by stabilising O-GlcNAcase (OGA) mRNA [156]. The loss of NAT10 arrests follicular development at the primary stage and results in premature ovarian insufficiency [157]. In early embryonic development, ac4C stabilises OCT4 mRNA, allowing embryonic stem cell self-renewal and pluripotency [158], and stabilises NOP2 mRNA to regulate the morula-to-blastocyst transition [159]. NAT10 is upregulated in gynaecologic malignancies, such as cervical and ovarian cancer, tying to poor prognoses, and promotes tumour cell proliferation and invasion through ac4C modification [160,161]. However, in animal models of premature ovarian insufficiency, elevated ac4C levels are tightly tied to ovarian microenvironmental damage [162]. Taken together, ongoing research on ac4C and related modifications points to the potential for RNA epitranscriptomic-based precision diagnostics and therapeutics in future gynaecologic clinical practice.

### 2.8. 5-Methoxycarbonylmethyl-2-Thiouridine

Moreover, 5-methoxycarbonylmethyl-2-thiouridine (mcm^5^s^2^U) is a tRNA modification found at the wobble uridine (U34) position in the anticodon loop. Its biosynthesis requires tRNA methyltransferase 9-like protein (TRM9L) and AlkB homologue 8 (ALKBH8) to work together [163,164,165]. In eukaryotes, mcm^5^s^2^U stabilises the translation reading frame, and its absence reduces A-site selection efficiency by the ribosome, causing +1 frameshift errors [166]. Studies illustrate that mcm^5^s^2^U cooperates with m^6^A, promoting ribosome A-site binding efficiency to inhibit m^6^A-mediated mRNA decay to control oncogenic mRNA stability [167,168]. Models of non-associative learning have proposed that elevated mcm^5^s^2^U levels correlate with the increased synthesis of polyglutamine proteins and more neuronal excitability, suggesting a possible role in neural plasticity [169].

Although the involvement of mcm^5^s^2^U in essential biological processes has been elucidated, the possibilities for mischief in female reproductive physiology and gynaecologic diseases have not been studied directly. Due to the crucial role it plays in the maintenance of translation fidelity and the regulation of cellular differentiation [167,168], further studies could explore the control mechanisms during embryonic development, oogenesis and cyclic endometrial changes. As associations between mcm^5^s^2^U, m^6^A and poor prognoses in breast cancer have been reported, a systematic study of this modification in the initiation and progression of gynaecologic tumours could provide traction for the development of new biomarkers and therapeutic targets [167,170].

### 2.9. Crosstalk Between RNA Modifications

RNA modifications form another layer of gene expression regulation. Studies show that various RNA modifications do not exist in isolation, but rather in complex interactive networks in cells. These interactions are primarily mediated through the coordinated activities of writers, erasers and readers, which collectively determine the fate of RNA at multiple levels, including splicing, stability, translation and localisation [171].

To establish a unified framework for understanding these diverse processes, we propose that RNA modifications in the female reproductive system operate as an integrated ‘Epitranscriptomic Code’. This framework consists of three hierarchical layers: the ‘Molecular Input Layer’, where diverse modifications (m^6^A, m^5^C, Ψ, etc.) are deposited by specific writers in response to hormonal and environmental signals; the ‘Regulatory Processing Layer’, where reader proteins interpret these marks to modulate the post-transcriptional life cycle of key reproductive transcripts; and the ‘Functional Output Layer’, where these coordinated changes culminate in physiological milestones such as oocyte maturation or pathological states similar to tumorigenesis. This multi-layered network ensures that female reproductive cells can achieve rapid, reversible and precise gene expression control, moving beyond isolated mechanisms to a systems-level regulatory logic [172].

In this context, the cooperative and antagonistic interactions between modification enzymes are particularly significant, as this dynamic is essential for fine tuning RNA activity. Mechanistically, such crosstalk can occur through several modes, including competition for overlapping or structurally constrained RNA regions, RNA structural remodelling induced by specific modifications and feedback regulation among modification enzymes [31,173].

For instance, ALKBH5 (m^6^A demethylase) and NSUN4 (m^5^C methyltransferase) are regulated mainly at the protein level. They mainly affect mRNA splicing, protein acetylation and ubiquitination. These two modification systems create post-transcriptional feedback loops through the mutual modification of each other’s effector transcripts, the ALKBH5 transcripts carrying m^5^C modifications and the NSUN4 transcripts carrying m^6^A sites. This type of reciprocal regulation represents a typical feedback mechanism that contributes to the maintenance of epitranscriptomic homeostasis.

The methyltransferases METTL14 and METTL16 and the m^5^C methyltransferases NSUN4 and NSUN5 co-regulate each other closely, related to mitochondrial functioning and post-translational processes such as phosphorylation and SUMOylation [174]. In addition, m6A writers, such as METTL3/METTL14, can regulate the expression of other RNA modification enzymes via the m6A-dependent control of RNA stability and translation, further strengthening the interconnected regulatory network [172].

Analysing transcriptomes with nanopore direct RNA sequencing, we found a negative correlation between m^6^A and pseudouridine (Ψ) within the same transcript, suggesting Ψ-enriched transcripts have fewer m^6^A sites. This observation may be explained by competition for structurally constrained regions or steric hindrances between modification machineries [175]. Inside polysomes, however, the two modifications display a strong synergism, jointly enhancing translation. Functionally, Ψ improves codon–anticodon stability and translation fidelity, whereas m6A promotes translation initiation, indicating a division of labour between these modifications [176]. In this hierarchy, Ψ contributes the most to translation efficiency over m^6^A [177].

The quantitative evaluation of RNA modifications has been enabled through mass spectrometry [178]. Knocking out METTL3 causes compensatory increases in Ψ levels in high m^6^A messages, which suggests m^6^A may inhibit Ψ installation. Conversely, the loss of the Ψ synthase TRUB1 unexpectedly decreases m^6^A levels, suggesting a reciprocal regulatory network between the two systems [177].

A-to-I editing refers to the process of converting adenosine (A) into inosine (I) catalysis by a family of enzymes known as the ADARs. Both methylation on adenosine and m^6^A modification occur, meaning that the two modifications are negatively correlated and often mutually exclusive in transcripts. Mechanistically, m6A disrupts the local RNA duplex structures required for ADAR binding, thereby inhibiting A-to-I editing [173]. The knockdown of METTL3 markedly increases global A-to-I editing, though FTO knockdown reduces A-to-I levels. These modifications show widespread antagonistic interactions [173,179].

In a study reporting on early-stage lung adenocarcinoma, an expression signature based on m^6^A- and m^5^C-related regulatory genes has strong prognostic capabilities [180]. Hepatocellular carcinoma (HCC) oncogenes, such as epidermal growth factor receptor (EGFR), are regulated in part by m^6^A and m^5^C modification to drive tumour progression. ALYREF-mediated m^5^C modification exerts a stabilising effect on EGFR mRNA, leading to the upregulation of EGFR expression [181]. In contrast, m^6^A seems to exert incremental effects at experimental concentrations. METTL3 enhances the translation efficiency of EGFR, while YTHDF2, through its binding to m^6^A sites, recruits degradation complexes that speed up the degradation of the EGFR transcript [182,183]. This illustrates a coordinated regulatory model in which m^5^C primarily stabilises transcripts, whereas m6A dynamically balances translation and decay [120].

In contrast, in glioblastoma (GBM), the m^6^A modification system and A-to-I editing have cell type-specific functions. In GBM stem cells (GSCs), METTL3 and METTL14 preserve m^6^A levels to inhibit self-renewal and tumorigenesis, while their downregulation facilitates GSC proliferation and stemness [184]. In differentiated GBM cells, METTL3 can enhance the presence of the ADAR1 protein via the m^6^A-YTHDF1 axis; in turn, ADAR1 binds to and stabilises cyclin-dependent kinase 2 (CDK2) mRNA, facilitating cell cycle progression and tumour growth [185]. These findings further support a context-dependent interaction between RNA modifications, where the same modification can exert distinct biological effects depending on the cellular state [172].

It has been demonstrated that genetic alterations, such as the PIK3CA mutation and PTEN loss, drive the activation of the phosphatidylinositol 3-kinase/protein kinase B/mammalian target of rapamycin (PI3K/Akt/mTOR) pathway in ovarian cancer [186]. Epitranscriptomic studies indicate that m1A modification can promote tumour progression via this pathway in gastric and bladder cancers. In addition, the m^6^A demethylase ALKBH5 modulates autophagy in ovarian cancer through the miR-7/EGFR axis [187]. These findings suggest that m^6^A, m1A, and other RNA modifications may provide an additional layer of regulation over the PI3K signalling pathway.

Outside cancer, modification crosstalk is also important in non-malignant diseases. In cellular senescence models, NSUN2-mediated m^5^C and METTL3/METTL14-mediated m^6^A cooperatively modify the 3′UTR of p21 mRNA, mutually promoting each other and enhancing p21 translation, a mechanism important for oxidative stress-induced ageing [188].

In patients with premature ovarian insufficiency (POI) and chemotherapy-induced POI models, ovarian granulosa cells show elevated m^6^A levels and reduced FTO expression, accompanied by impaired proliferation and increased apoptosis [189]. Since oxidative stress is a key pathological factor in POI, multiple insults can induce excessive ROS accumulation in granulosa cells, leading to mitochondrial dysfunction and follicle depletion [190,191]. Because of the close relationship between oxidative stress and cellular senescence, Li et al. showed that m^6^A/m^5^C cooperative modification regulates p21 to influence cell ageing, a mechanism likely relevant in granulosa cells from POI patients. Therefore, the dysregulation of m^6^A/m^5^C crosstalk may contribute to the ovaries’ functional decline. Therefore, the dysregulation of RNA modification crosstalk may represent an important epitranscriptomic mechanism underlying ovarian ageing and functional decline.

Taken together, these findings show that complex networks of RNA modifications play important roles in disease development and progression. However, several key questions remain unresolved, including the temporal hierarchy of modification deposition, the structural determinants governing modification interplay and the quantitative contribution of each modification to gene regulation. Addressing these issues will require integrative approaches that combine high-resolution sequencing, mass spectrometry and functional epitranscriptomic editing technologies.

## 3. Functional Mechanisms of RNA Modifications in Female Reproductive Physiology

### 3.1. Oogenesis and the Ovarian Follicle Microenvironment

Oogenesis is a controlled biological process that starts with primordial germ cells during embryogenesis and progresses through mitosis to meiosis and growth to produce mature oocytes or eggs that can be fertilised. In addition, the development of multi-celled embryos can also support [192]. In mammals, the growing oocyte undergoes extensive transcription and accumulation of RNA molecules known as maternal factors that continue until oocyte growth, meiotic maturation and the early period after maturation. During classification methylation, when fully meiotically matured oocytes are transcriptionally silent, these maternal mRNAs are used for protein synthesis as the primary source of genetic information [157,193,194]. Upon entering meiotic maturation, fully grown oocytes exhibit very little transcriptional activity [195]. Hence, oocyte quality and the ability of early embryos to develop depend less on the new transcription of genes than the spatio–temporal control of pre-stored maternal RNAs in the oocyte, particularly their stability, localisation, translated activation and final degradation [196,197]. The development of oocytes does not happen alone; instead, it runs with the development of granulosa cells (GCs) in the microenvironment prevalent in the ovarian follicle. In addition, RNA modifications have significant control functions [198,199].

#### 3.1.1. Differentiation of Primordial Germ Cells into Oocytes

The proximal epiblast gives rise to primordial germ cells (PGCs), which subsequently migrate to and settle in the genital ridge, initiating oogenesis [200,201]. RNA modifications and their regulatory factors play important roles during this early colonisation phase; m^6^A modification controls normal meiotic progression in the female germline through its reader protein YTHDC2. The loss of YTHDC2 causes meiotic arrest in germ cells and leads to follicle loss and infertility in adult ovaries, a function that depends on RNA helicase activity and the ability to recognise m^6^A modifications [27,202,203]. During the leptotene stage of meiosis, ac4C modification and its writer protein NAT10 are necessary. Nat10 deletion driven by Stra8-Cre causes follicle arrest at the primary stage and premature ovarian failure. Chromosome spread immunostaining shows an increased proportion of leptotene-like oocytes and a sharp reduction in zygotene oocytes, indicating meiotic arrest at the leptotene stage [156,157].

As embryonic development moves to late stages after 16.5 days post-coitum, m^6^A levels in the ovary slowly drop, reaching their lowest point at birth and rising after birth. Correspondingly, nuclear expression of the m^6^A demethylase FTO increases markedly during germ cell nest breakdown. FTO controls the alternative splicing of genes such as cyclin-dependent kinase 5 (CDK5), affecting cell cycle progression and, thus, controlling the timing of nest breakdown and the efficiency of primordial follicle assembly, finally affecting the size of the primordial follicle pool [204]. During this critical window of primordial follicle assembly, P-body-like granules also play an important role [205]. The m^6^A reader protein YTHDF2 promotes P-body assembly by recruiting core P-body proteins. The increased expression or enhanced function of YTHDF2 inhibits germ cell nest breakdown and primordial follicle formation. These granules are present throughout the formation of primordial follicles and are distributed in the cytoplasm of oocytes during the cyst and primordial follicle stages [206].

After birth, when oocytes enter the growth phase, m^5^C modification starts to work early in folliculogenesis. The m^5^C reader protein YBX1 stabilises m^5^C-modified RNA and functions by recruiting the poly(A)-binding protein PABPC1A and setting up the molecular basis for subsequent oocyte maturation [207,208]. Meanwhile, NSUN5 mediates the m^5^C modification of rRNA and is necessary for maintaining maternal RNA [121]. As oocytes move from the GV stage to the MII stage, m^5^C modification slowly builds, peaking at MII and then declining markedly during early embryonic development [209].

#### 3.1.2. Oocyte Meiosis

Meiotic maturation is a key step for oocytes to acquire fertilisation competence, with multiple RNA modifications taking part in complex regulatory interactions. Under transcriptionally silent conditions, m^6^A modification shows the dual control of the maternal RNA metabolism. METTL3-installed m^6^A marks are recognised by YTHDF2, which promotes maternal RNA degradation through the CCR4-NOT deadenylation complex [78,206]. METTL3 can also enhance the stability of target mRNAs, such as MYC, through interaction with IGF2BP3. The Vir-like m^6^A methyltransferase-associated protein KIAA1429 also takes part in this process [28,210].

During the transition from the Germinal Vesicle (GV) stage to the Metaphase II (MII) stage, ALKBH5 selectively removes m^6^A marks from specific maternal RNAs, known as loss-GMD transcripts. This demethylation is needed for maternal RNA clearance. The loss of ALKBH5 causes persistent m^6^A marks and abnormal transcript accumulation mediated by IGF2BP2 activity [211]. During transcriptionally active periods, m^6^A interacts with YTH domain-containing protein 1 (YTHDC1) and splicing regulators, such as serine/arginine-rich splicing factor 3 (SRSF3) and SRSF7, coordinating precursor mRNA splicing and alternative polyadenylation to produce the functionally mature transcripts required for meiotic maturation [212]. The m^6^A modification collaborates with histone methylation to regulate chromatin condensation and spindle assembly, which affects accurate chromosome segregation during meiosis [213,214,215,216].

In addition to m^6^A, ac4C modification is required for the initiation of meiotic maturation competence. The Zp3-Cre-mediated conditional deletion of N-acetyltransferase 10 (NAT10) occurs at the primary follicle stage but subsequently brings about meiotic arrest and disrupts the morphological transition of oocytes from the Non-surrounded Nucleolus (NSN) type to the Surrounded Nucleolus (SN) type. NAT10 is implicated in the control of nuclear maturation capacity through processes that include poly(A) tail shortening and the degradation of maternal RNA, involving CCR4-NOT transcription complex subunit 6-like (CNOT6L), CNOT7 and the action of B-cell translocation gene 4 (BTG4) [157]. NAT10 maintains the stability of O-GlcNAcase (Oga) mRNA and further regulates oocyte maturation [156].

#### 3.1.3. Granulosa Cell Function and the Follicular Microenvironment

Granulosa cells (GCs) are an important functional unit of the follicle microenvironment. GCs form metabolic syncytia with the oocyte via gap junctions and supply energy substrates, steroid hormones and nutritional support. The accumulation of ageing-related oxidative stress, mitochondrial dysfunction and genomic instability triggers apoptosis and functional decline in GCs, while imbalances in post-translational modification networks worsen secretion defects and cell cycle arrest [217,218]. Because of the central control role that GCs play in follicle development, targeted interventions in their metabolic state, redox balance and epigenetic modifications offer accurate treatment strategies to delay ovarian ageing and improve assisted reproductive outcomes. In the control of GC proliferation, m^6^A modification shows dual effects by accurately controlling the stability of cell cycle-related transcripts. FTO-mediated demethylation maintains the stability of cyclin D1 (CCND1) mRNA, and its loss leads to CCND1 degradation via YTHDF2, causing cell cycle arrest and suppressed GC proliferation [219]. Conversely, under brain-derived neurotrophic factor (BDNF) stimulation, YTHDF2 expression is upregulated via the extracellular signal-regulated kinase (ERK) pathway, promoting the expression of key cycle regulators such as CDK4 and PCNA, thus driving GC proliferation [220].

M^6^A modification also plays a role in balancing autophagy and apoptosis in GCs. During follicle atresia, the downregulation of METTL3 reduces m^6^A levels, leading to the increased expression of autophagy-related ULK1 mRNA and an elevated LC3II/LC3I ratio, finally activating GC autophagy and promoting follicle degeneration [221]. FTO maintains the stability of the anti-apoptotic factor MNT mRNA through demethylation and activates the AKT/Nrf2 pathway to reduce oxidative stress-induced apoptosis [222].

In response to oxidative stress and ageing, GCs rely on the m^6^A modification network to maintain cellular balance. FTO expression drops with ovarian ageing, causing the accumulation and impaired degradation of m^6^A-modified ageing-related transcripts such as FOS mRNA, speeding up GC senescence [223]. In addition, IGF2BP1 expression decreases under oxidative stress, impairing its m^6^A-dependent stabilisation of MDM2 mRNA, which in turn reduces GC viability and disrupts cell cycle regulation [224]. FTO also stabilises exosomal circular RNA circBRCA1, reducing oxidative stress-induced mitochondrial damage via the miR-642a-5p/FOXO1 axis [225]. In steroidogenesis, FTO interacts with the androgen receptor (AR) to take part in hormone synthesis regulation in GCs, although the exact molecular mechanism remains to be clarified [226].

Under pathological conditions, m^6^A dysregulation is tightly linked to polycystic ovary syndrome (PCOS) and POI. In GCs from PCOS patients, the upregulation of FTO reduces m^6^A levels on FLOT2 mRNA and enhances its stability, promoting GC proliferation, inhibiting apoptosis and inducing insulin resistance, finally leading to GC dysfunction [227]. Other studies indicate that the reduced m^6^A modification of FOXO3 mRNA causes abnormally increased expression, disrupting GC homeostasis [228]. In the pathogenesis of POI, GCs show markedly reduced FTO expression and globally increased m^6^A levels, leading to disrupted cell cycle regulation and heightened apoptotic sensitivity. Transcriptomic analysis displays a marked increase in m^6^A-modified genes in GCs from patients with diminished ovarian reserve. The FoxO signalling pathway, adherens junctions and actin cytoskeleton regulation are enriched in differentially methylated genes. Out of the mutations identified, 58 of the genes, such as BUB1B, TOP2A and PHC2, have dual abnormality in m^6^A modification and expression, which initiates decreased GC proliferation and defective meiosis [229]. Environmental toxins such as cyclophosphamide can increase m^6^A levels in GCs in a time-dependent fashion. Accompanied by the inhibition of demethylase FTO, the reader proteins YTHDF1/2 and YTHDC1/3 disrupt m^6^A homeostasis, accelerate GC apoptosis and deplete the ovarian reserve [230].

Outside m^6^A, m^5^C modification and its regulatory enzymes also contribute to GC pathology. In GCs from PCOS patients, m^5^C mediated by NOP2 or NSUN7 is markedly upregulated, stabilising NLRP3 mRNA, activating the NLRP3 inflammasome, promoting caspase-1 cleavage and gasdermin D processing and finally driving GC pyroptosis [231]. Conversely, in POI, m^5^C modifications exert protective effects through different control axes. For example, the YBX1 protein from human umbilical mesenchymal stem cell-derived exosomes stabilises COX5B mRNA by recognising TRDMT1-mediated m^5^C modification, reducing oxidative stress-induced GC senescence [232].

### 3.2. Fertilisation, the Maternal to Zygotic Transition and Early Embryonic Development

Early embryonic development after fertilisation involves a shift from the reliance on maternal RNA to the activation of transcription from the embryonic genome. This transition is accompanied by the extensive reprogramming of gene expression. RNA modifications are an important layer of epitranscriptomic regulation and have important functions in controlling early post-fertilisation transcriptional activation and gene expression.

Research shows that m^6^A changes dynamically during the maternal-to-zygotic transition (MZT). It can be inherited from maternal mRNA and can be added to transcripts synthesised after fertilisation, supporting regulation in two directions. That is, m^6^A promotes the decay of a subset of maternal mRNAs, allowing the removal of transcripts that are no longer required. Conversely, m^6^A stabilises a smaller group of mRNAs and enhances their translation, supporting the processes needed for embryonic development. In mice, m^6^A-associated factors such as Ythdc1 and Ythdf2 serve as reader proteins during preimplantation development and assist MZT [233]. In early mammalian embryos, m^6^A also influences RNA chromatin interactions and reshapes transcriptional control networks, modulating embryonic genome activation and cell fate decisions [234].

During the oocyte-to-embryo transition in mice and humans, m^6^A and N6,2′-O-dimethyladenosine (m^6^Am) show species-specific regulatory patterns. Zygotic genome activation (ZGA) marks a major control point during which the m^6^A and m^6^Am methylation landscape changes greatly. Transcripts with these marks are typically more abundant and more translated. Moreover, m^6^A appears to play a dual role in transposable element RNA regulation by promoting its activity before ZGA and curbing runaway activity during ZGA [235]. M^6^A is present in pig embryos from the one-cell stage to the blastocyst stage with an upward shift from the morula to blastocyst stage. Inhibiting m^6^A methylation causes developmental arrest, abnormal lineage allocation and increased apoptosis and autophagy at the blastocyst stage, displaying that m^6^A is needed for normal development [236]. Y-box binding protein 1 (YBX1) influences the ZGA and histone modification states in pig embryos by regulating the expression of METTL3 and IGF2BP1 [237]. Single-cell transcriptomic analyses have also characterised the gene expression dynamics of MZT in early pig embryos [238].

In addition, m^5^C helps maintain maternal RNA stability and supports the transition to ZGA. The m^5^C methylation produced by NSUN6 may add a distinct layer of cell division control during human embryonic development [209]. The m^5^C binding protein ALYREF promotes mRNA export from the nucleus to the cytoplasm. YBX1 helps secure orderly MZT by stabilising maternal RNAs marked by m^5^C. YBX1 is linked to translational repression in zebrafish embryos, whereas in mammals, it may also take part in ZGA through an m^6^A-related route [45,207].

Additionally, ac4C plays a part in the key stages of embryonic development. The ac4C writer NAT10 is needed for ZGA and blastocyst formation in mouse embryos. The loss of NAT10 leads to arrest at the two-cell or morula stage and disrupts both maternal mRNA decay and ZGA, showing that ac4C assists with development by regulating mRNA stability and transcriptional activity [239]. Mechanistic work further suggests that NAT10 modulates ac4C on specific targets, such as Nop2, thus altering mRNA stability and protein production. By these means, NAT10 influences cell fate decisions and promotes the transition from the morula to the blastocyst [159].

A-to-I RNA editing is another RNA modification that assists with early embryonic development. Single-cell transcriptomic analyses indicate that editing events in early human embryos are strongly stage specific. At the eight-cell stage, editing is extensively reprogrammed, and editing levels drop at most sites. Editing also tends to occur at splice sites of lncRNAs, and editing levels tie closely to lncRNA splicing indices, pointing to its function in transcriptional control through the modulation of lncRNA splicing [240]. Outside transcription-related effects, A-to-I editing may also influence maternal RNA stability after fertilisation by shifting codon usage patterns and, thus, promoting maternal RNA clearance. For example, inosine modifications in oocyte transcripts are enriched at wobble positions within coding regions and are markedly reduced when CCR4-NOT transcription complex subunit 6 like (Cnot6l) is absent. These results give credence to the idea that A-to-I editing alters codon usage in a manner favourable for maternal RNA stability and MZT [104,241,242].

In embryos, epigenetic mechanisms, such as histone modifications and DNA methylation, aid in genome reprogramming and transcriptional activation [243,244]. The embryonic genome becomes activated efficiently through RNA modifications and related mechanisms, allowing cells to develop proper fates. Regulatory mechanisms for RNA modifications differ in their species and cell type specificity, demonstrating that such mechanisms are subject to the species context and developmental stage.

When taken as a whole, RNA modifications have a powerful effect on fertilisation and embryonic genome activation at multiple levels, including RNA stability, splicing, translation and transcriptional control. The interplay between gene and chromatin dynamics not only reflects developmental competence but also enables the coordination between histone modifications] and DNA methylation to achieve the timing and efficiency of ZGA. By shaping genome activation, RNA stability and cell cycle regulation, RNA modifications are needed for essential early developmental processes. The disruption of these pathways can lead to developmental arrest and implantation failure and can impair lineage differentiation and overall embryo quality. Further work could define the stage-specific functions of different RNA modifications and their regulators across embryonic development to identify new targets and strategies for diagnosing and treating developmental disorders.

### 3.3. Establishment of Uterine Receptivity

Endometrial receptivity is a prerequisite for successful embryo implantation and the maintenance of pregnancy. Its establishment depends on tightly controlled ovarian steroid hormone signalling and the dynamic remodelling of the epitranscriptome [245]. During normal pregnancy, m^6^A levels in the endometrium increase progressively, and the spatial distribution of related regulators changes in a gestational stage-dependent manner. This balance is disrupted in infertility, with reduced transcript levels of METTL16 and WTAP, abnormally elevated ALKBH5 and decreased IGF2BP2, changes that are tied to the dysregulation of immune-related signalling pathways [246].

Between days 16 and 25 of pregnancy, METTL3 is highly expressed in luminal and glandular epithelial cells of the endometrium, and its expression can be induced by oestrogen and progesterone. METTL3 supports CTGF expression by keeping m^6^A on CTGF mRNA, which promotes endometrial epithelial proliferation and helps create an endometrial environment that allows implantation [247]. Further studies report that METTL3 is markedly reduced in the endometria of patients with recurrent implantation failure (RIF) and stage-IV endometriosis (EMs)-associated infertility. METTL3 levels correlate positively with progesterone receptor (PGR) and MYC levels and correlate negatively with ELF3. A uterus-specific conditional knockout of Mettl3 driven by Pgr-Cre shows that the loss of METTL3 leads to complete implantation failure and female infertility. Mechanistically, METTL3-dependent m^6^A promotes the degradation of oestrogen-responsive transcripts such as Elf3 and Celsr2 by targeting their three prime untranslated regions, thus limiting excessive oestrogen signalling. Simultaneously, METTL3 maintains the expression of PGR and its downstream target Myc, supporting normal progesterone responsiveness. This coordinated balance between oestrogen and progesterone receptor pathways is needed for uterine receptivity and for decidualisation, thus supporting female fertility at the organism level [248]. Recent work further indicates that the loss of METTL3 induces SUV39H1-mediated histone H3 lysine 9 trimethylation (H3K9me3) across the gene body of Sequestosome 1 (SQSTM1, also known as P62). Meanwhile, METTL3 deficiency stabilises P62 mRNA in an m^6^A-dependent manner. These changes lead to P62 accumulation, the activation of autophagy and inflammatory programmes and a marked reduction in embryo adhesion. Mice that are conditional knockouts for Mettl3 in their uteruses display increased P62, tumour necrosis factor-α (TNF-α) and the abnormal development of endometrial glands, which leads to implantation failure [249].

At the mechanistic level, METTL3 directly installs m^6^A on progesterone receptor (PGR) mRNA and enhances the translation of PGR protein in a YTHDF1-dependent manner. This process is crucial for the in vitro decidualisation of endometrial stromal cells [250]. Nonetheless, METTL3’s impact is context dependent. In recurrent implantation failure (RIF) patients, pathological increases in METTL3 expression and global m^6^A levels in the endometrium are associated with decreased receptivity. In this context, increased METTL3 enhances m^6^A on homeobox A10 (HOXA10) mRNA and accelerates its decay, thus reducing β3 integrin expression. This change is accompanied by the abnormal activation of empty spiracles’ homeobox 2 (EMX2) signalling and finally impairs implantation [251]. Unlike the complex role of METTL3, the loss of METTL14 drives implantation failure through an alternative route. METTL14 deficiency leads to the abnormal activation of oestrogen receptor alpha (ERα) signalling and ERα phosphorylation and is accompanied by the pathological activation of innate immune pathways. These changes disrupt immune tolerance at the maternal foetal interface and lead to implantation failure [252].

Outside m^6^A, m^5^C also contributes to the regulation of endometrial receptivity. The m^5^C methyltransferase NSUN2 is highly expressed in epithelial cells during the proliferative phase. Its overexpression markedly reduces embryo attachment and is linked to recurrent implantation failure. Mechanistically, NSUN2 installs m^5^C on claudin 4 (CLDN4) mRNA and suppresses Sirtuin 4 (SIRT4), which increases histone H3 lysine 9 acetylation (H3K9ac) and jointly elevates CLDN4 expression. NSUN2 is also reported to promote exon-skipping events in the signal transducer and the activator of transcription 1 (STAT1) and matrix metalloproteinase 14 (MMP14). These alterations impair endometrial receptivity [122].

In conjunction, m^6^A and m^5^C RNA modifications work through major regulators such as METTL3, METTL14 and NSUN2 to coordinate the establishment and maintenance of endometrial receptivity at the post-transcriptional level. These enzymes influence hormone receptor signalling, cell adhesion, splicing and the immune microenvironment to support successful implantation. Imbalance in the RNA modification network, whether excessive activity or loss of function, can lead to impaired receptivity and infertility-associated pathology. These findings show the importance of maintaining a dynamic epitranscriptomic balance for normal reproductive outcomes.

### 3.4. Placental and Embryonic Development

In early mammalian embryogenesis, m^6^A supports successful MZT by regulating the timing of maternal messenger RNA clearance and the activation of the zygotic genome. The loss of METTL3-mediated m^6^A in mouse oocytes impairs maternal mRNA decay, delays ZGA and leads to developmental arrest or embryonic lethality [253]. Simultaneously, YTHDF2 promotes the removal of maternal transcripts by recognising m^6^A, a process needed for early zebrafish development and for maternal mRNA clearance in goat embryos [254,255]. In addition, IGF2BP2 serves as a key maternal activator at the two cell stages by regulating specific targets that support blastocyst formation, whereas IGF2BP3 keeps maternal RNA stable to sustain early zebrafish development [256,257]. Outside m^6^A, m^5^C assists with preimplantation development. The m^5^C methyltransferase NSUN5 (NOL1/NOP2/Sun domain family member 5) is greatly upregulated at the two-cell stage, and its loss reduces blastocyst formation, lowers cell numbers and increases apoptosis. At the mechanistic level, NSUN5 regulates the expression of Hippo pathway components LATS1 (large tumour suppressor 1) and LATS2, affecting YAP1 (yes-associated protein 1) nuclear localisation and the expression of the trophoblast marker CDX2. Accordingly, NSUN5 supports the proper segregation of the inner cell mass and trophoblast lineages [258].

In trophoblast stem cells, m^6^A has dual effects on pluripotency and differentiation. METTL3-mediated m^6^A can reduce the stability of pluripotency factor mRNAs and promote their decay, allowing the transition of mouse embryonic stem cells (mESCs) from a naive state towards differentiation [259]. The loss of METTL3 impairs the self-renewal of mESCs and leads to embryonic lethality [260]. Likewise, METTL14 deficiency markedly delays post-implantation development and impairs the maturation of ectoderm-derived lineages [261]. At the rRNA level, METTL5 installs m^6^A on 18S rRNA and influences the differentiation potential of mESCs. METTL5 loss leads to defects in germ layer differentiation and the abnormal accumulation of c-MYC protein [262]. METTL16 could also influence early development by regulating methionine adenosyltransferase 2A (MAT2A) mRNA abundance [263]. YTHDC1 helps mature oocytes through the regulation of alternative polyadenylation and splicing, while YTHDC2 supports meiosis and embryonic development by modulating mRNA translation efficiency and stability [27,212].

As placental development proceeds, m^6^A plays a key role in trophoblast invasion, migration and the maintenance of functions at the maternal–foetal interface. In placental tissue, m^6^A sites are enriched in the three prime untranslated regions and in coding sequences near the stop codon. Abnormal m^6^A levels are tightly linked to foetal growth restriction and preeclampsia, and placental FTO expression is also tied to foetal growth [264], while placental FTO expression levels also correlate with foetal growth [265]. METTL3 enhances trophoblast invasiveness by methylating myosin light chain kinase (MYLK) mRNA, an effect needed for normal placental functioning [266]. YTHDC1 assists with placental homeostasis by promoting the degradation of circMPP1 through pathways involving nuclear factor kappa B (NF-κB) and mitogen-activated protein kinase 3 (MAPK3) signalling [267]. Differences in m^6^A levels within the five prime untranslated regions are tied to foetal weight, suggesting that translation-related mechanisms assist with placental functioning [264].

Demethylases exhibit different functional patterns during placental development. Placental FTO expression correlates positively with newborn birth weight and could help regulate foetal growth [265]. ALKBH5 regulates trophoblast invasiveness by demethylating cysteine-rich angiogenic inducer 61 (CYR61) mRNA. Elevated ALKBH5 expression has been tied to recurrent miscarriage [268]. ALKBH5 is also involved in placental regulation by modulating the m^6^A modification of the mRNA of the peroxisome proliferator-activated receptor gamma (PPARG) in preeclampsia [269]. FTO can additionally impact the growth and differentiation of embryonic stem cells through the demethylation of long interspersed nuclear element 1 (LINE1) RNA [270]. Aside from methylation and demethylation, A-to-I RNA editing also contributes to placental biology. In the placenta, endogenous double-stranded RNA (dsRNA) is modified by the editor ADAR1 to maintain innate immune homeostasis. The loss of ADAR1 leads to heightened interferon signalling and activates an integrated stress response (ISR) in junction zone trophoblasts, affecting placental function and leading to lethality. The phenotype in question is associated with the accumulation of double-stranded RNA derived from the three-prime untranslated region(s) of interferon-stimulated genes (ISGs), suggesting the important role of RNA editing in regulating immunity at the maternal–foetal interface [271].

**Figure 2:**
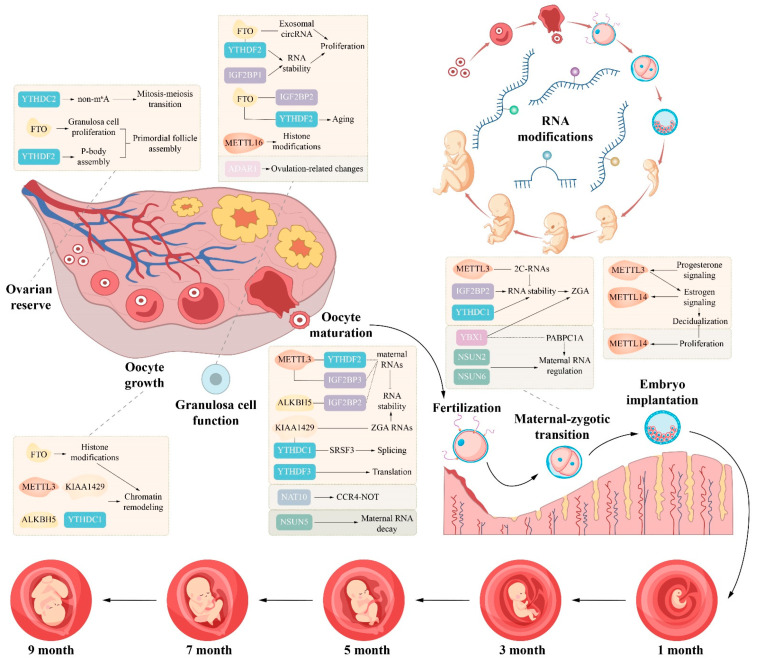
Regulatory roles and molecular mechanisms of RNA modifications in female reproductive physiology. Ovarian reserve and oocyte growth: Epitranscriptomic regulators (e.g., FTO, YTHDF2, and METTL3/KIAA1429) modulate primordial follicle assembly and oocyte growth through mechanisms involving histone modifications, chromatin remodelling, and P-body assembly. Granulosa cell function and oocyte maturation: In granulosa cells, modifications influence proliferation and ageing via the FTO/IGF2BP2/YTHDF2 axis. During oocyte maturation, “writers” and “readers” (e.g., METTL3, ALKBH5, YTHDC1) coordinately regulate maternal RNA stability, splicing, and translation to ensure oocyte quality. Fertilisation and Maternal-Zygotic Transition (MZT): Following fertilisation, RNA modifications (including m6A and m5C) are essential for the degradation of maternal RNAs and the activation of the zygotic genome (ZGA). Key factors such as YTHDF3 and NSUN5 facilitate this transition by controlling RNA decay and translation. Embryo implantation and pregnancy: m6A regulators (METTL3/14) modulate progesterone and oestrogen signalling pathways to promote decidualization and successful embryo implantation. The progression from the first month to full-term pregnancy (9 months) relies on the precise spatio-temporal control of these RNA modifications to support foetal development.

Taken together, RNA modifications form a multi-level regulatory network that spans the reproductive cycle, including oogenesis, fertilisation, embryonic development, the establishment of uterine receptivity and placental formation (Figure 2). From primordial germ cell differentiation to MZT and from shaping endometrial receptivity to maintaining immune homeostasis at the maternal foetal interface, m^6^A, m^5^C, ac4C and A-to-I RNA editing work together. By controlling transcript stability, translation efficiency and the timing of RNA decay, these mechanisms secure the accurate control of timing and location for germ cell fate decisions and embryonic development. The disruption of these epitranscriptomic pathways is tightly linked to obstetric and gynaecologic disorders, including polycystic ovary syndrome, premature ovarian insufficiency, recurrent implantation failure and preeclampsia. It offers a basis for understanding the molecular causes of reproductive dysfunction.

Given the critical role of these RNA modifications in maintaining normal reproductive physiology, it is not surprising that their dysregulation is increasingly implicated in a spectrum of obstetric and gynaecologic disorders. The following sections will explore how these molecular aberrations contribute to the pathogenesis of such diseases, from gynaecologic cancers to pregnancy-related complications.

## 4. RNA Modification Regulation in Gynaecologic Cancers

**Figure 3:**
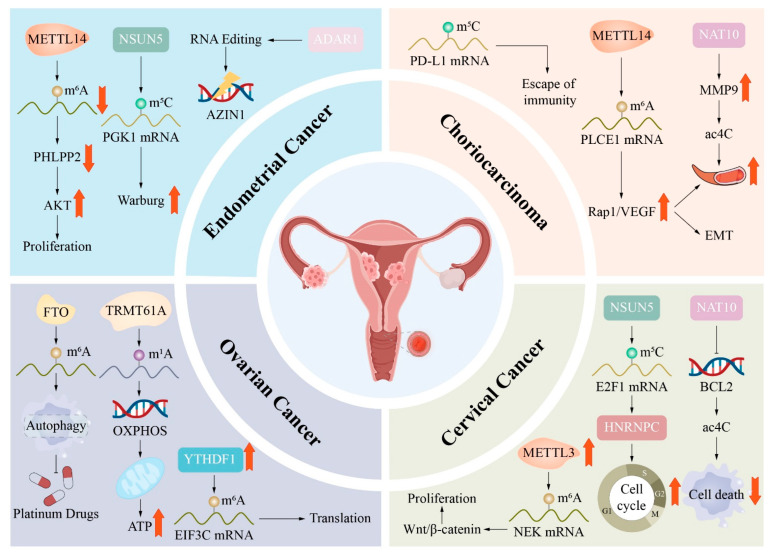
Dysregulation and oncogenic mechanisms of RNA modifications in gynaecological cancers. Endometrial Cancer: METTL14 (e.g., R298P mutation) leads to a global reduction in m6A levels, downregulating the AKT inhibitor PHLPP2 and subsequently hyperactivating AKT signalling. NSUN2-mediated m5C modification of PGK1 mRNA promotes the Warburg effect, while ADAR1-mediated A-to-I editing of AZIN1 alters coding sequences to drive oncogenesis. Choriocarcinoma: The METTL14/m6A/PLCE1 axis activates Rap1/VEGF signalling to drive Epithelial–Mesenchymal Transition (EMT) and potent angiogenesis. NAT10 promotes invasion by enhancing MMP9 mRNA stability via ac4C modification. Furthermore, m5C modification of PD-L1 mRNA facilitates immune escape by bypassing maternal immune surveillance. Ovarian Cancer: Genomic amplification of YTHDF1 promotes the translation of m6A-modified EIF3C mRNA to fuel tumour growth. TRMT61A-mediated m1A modification enhances oxidative phosphorylation (OXPHOS) and mitochondrial metabolism. Conversely, the demethylase FTO erases m6A marks on autophagy-related genes, promoting platinum resistance by facilitating the clearance of drug-induced cellular damage. Cervical Cancer: The METTL3/m6A/NEK2 axis activates the Wnt/β-catenin signalling pathway to inhibit apoptosis and promote proliferation. NSUN2-mediated m5C modification of E2F1 mRNA, recognised by ALYREF, facilitates nuclear-to-cytoplasmic export and cell cycle progression. Additionally, NAT10-mediated ac4C modification of BCL2 mRNA enhances its stability, contributing to chemoresistance by evading programmed cell death.

Gynaecologic malignancies include cervical, ovarian and endometrial cancers. They also include gestational trophoblastic malignancies, such as choriocarcinoma, and are a major group of cancers that threaten women’s health worldwide. Across pathological subtypes, these diseases show marked heterogeneity in causes, biological behaviours, clinical management and outcomes, which makes standardised care and precision treatment [272]. Recently, RNA modification systems, such as m^6^A, have become an important area for addressing these challenges. These reversible epitranscriptomic marks are regulated by networks of methyltransferases, demethylases and reader proteins that control major cancer-promoting and tumour-suppressing pathways at the post-transcriptional level. Disrupted RNA modification programmes can drive cancer progression in endometrial, ovarian and cervical cancers, and their expression patterns may work as biomarkers for early detection and prognostic assessment. Small-molecule inhibitors that target RNA modification regulators are being tested in clinical trials and could provide new options for precision therapy in gynaecologic cancers [273] (Figure 3).

### 4.1. Cervical Cancer

Cervical cancer (CC) remains a major cause of cancer-related death among women worldwide, and its development is tightly linked to persistent infections with high-risk human papillomavirus (HPV) types [274,275]. M^6^A is a key regulator of CC progression because it shapes the expression of cancer-promoting and tumour-suppressing genes. Among writers, METTL3 is greatly upregulated in CC tissue and is tied to a poor prognosis. METTL3 enhances the stability of target transcripts such as apoptotic chromatin condensation inducer 1 (ACIN1) and cathepsin L (CTSL) in an m^6^A-dependent manner, thus promoting the proliferation, invasion and migration of tumour cells [276,277]. Cell division cycle 25B (CDC25B), a key regulator of the G2/M phase transition, is recognised by YTHDF1 after its mRNA is modified by METTL3, which promotes translation and accelerates cell cycle progression [278]. In addition, METTL3 can regulate the m^6^A modification levels of targets, such as nuclear receptor subfamily 4 group A member 1 (NR4A1) and thioredoxin domain-containing protein 5 (TXNDC5), thus promoting malignant tumour behaviour by facilitating NR4A1 degradation and enhancing TXNDC5 stability, respectively [279,280]. METTL14 is also highly expressed in CC and increases the stability of tripartite motif-containing 11 (TRIM11) mRNA through m^6^A, which promotes tumour progression by activating AKT signalling via the ubiquitination of the phosphatase PHLPP1 [281]. ZC3H13 regulates centromere protein K (CENPK) and cytoskeleton-associated protein 2 (CKAP2) through m^6^A. The ZC3H13 CENPK axis supports cancer stem-like traits and chemotherapy resistance, while the ZC3H13 CKAP2 axis mainly enhances proliferation, invasion and migration [273,282]. RNA-binding motif protein 15 (RBM15) is elevated in HPV-positive cervical squamous cell carcinoma and adenocarcinoma and promotes proliferation, migration and metastasis by modulating m^6^A on targets including Otubain 2 (OTUB2) and decorin (DCN) [283,284].

Among m^6^A readers, YTHDF1 is highly expressed in CC and promotes the translation of RANBP2 mRNA by recognising its m^6^A sites. This effect enhances tumour cell proliferation, migration and invasion through the regulation of RAN GTPase activity [285]. YTHDF2 reduces the stability of axis-inhibition protein 1 (AXIN1) mRNA, thus promoting the epithelial–mesenchymal transition (EMT) and chemotherapy resistance [286]. YTHDF3 is elevated in radioresistant CC cells and contributes to homologous recombination repair by enhancing the translation of RAD51 homologue D (RAD51D), which supports the resistance to radiotherapy [287]. Members of the IGF2BP family are often upregulated across cancers. In CC, IGF2BP3 serves as an oncogene and stabilises cancer-promoting transcripts, such as MYC, by binding m^6^A. IGF2BP2 can bind m^6^A-marked circARHGAP12 and increase the stability of forkhead box protein M1 (FOXM1) mRNA, promoting CC progression [28,87,288]. HPV E6 and E7 oncoproteins can increase IGF2BP2 expression to stabilise host MYC mRNA and support aerobic glycolysis. In addition, the viral E7 transcript, itself, depends on METTL14-mediated m^6^A and recognition by IGF2BP1 to maintain stability [289,290].

Among m^6^A demethylases, FTO is overexpressed in CC. By reducing m^6^A on cancer-promoting transcripts, such as E2F transcription factor 1 (E2F1) and MYC, FTO enhances their translation and promotes tumour cell proliferation and migration [291]. FTO can also reduce m^6^A on β-catenin mRNA to increase its expression, which elevates the activity of excision repair cross-complementation group 1 (ERCC1) and contributes to the resistance to radiotherapy and chemotherapy [292]. Conversely, ALKBH5 is often described as tumour suppressive in CC, and low ALKBH5 levels are tied to a poor prognosis. ALKBH5 can suppress glycolysis by reducing m^6^A on pyruvate dehydrogenase kinase 4 (PDK4) mRNA and can affect the lipid metabolism by promoting the decay of sirtuin 3 (SIRT3) mRNA [293,294]. However, in HPV E7-driven CC, ALKBH5 has also been reported to stabilise p21-activated kinase 5 (PAK5) mRNA through m^6^A-dependent demethylation and, thus, promote tumour progression. ALKBH5 expression also differs substantially between HPV-positive and HPV-negative cells [295].

Outside m^6^A, the role of m^5^C in CC is receiving increasing attention. NSUN2, a major m^5^C methyltransferase, is greatly upregulated in CC tissue. NSUN2 installs m^5^C on keratin 13 (KRT13) and leucine-rich repeat-containing 8A (LRRC8A) mRNAs, enhances their binding to the reader YBX1 and increases transcript stability, promoting tumour cell proliferation, migration and invasion [124,125]. Evidence for m1A regulators in CC is more limited. However, tRNA methyltransferase 10C (TRMT10C) is reported to be highly expressed and associated with a poor prognosis. Silencing TRMT10C suppresses tumour cell proliferation and migration [146].

M^6^A on ncRNAs also contributes to CC progression. After METTL3-mediated m^6^A, lncRNA FOXD2-AS1 becomes more stable and promotes tumour progression by recruiting lysine-specific demethylase 1 (LSD1) and suppressing p21 expression [84]. ZFAS1 works in an m^6^A-dependent manner to sponge miR-647 and relieve the repression of its downstream targets [296]. KCNMB2-AS1 and IGF2BP3 form a positive feedback loop. KCNMB2-AS1 increases IGF2BP3 expression through a ceRNA mechanism, while IGF2BP3 stabilises KCNMB2-AS1 in an m^6^A-dependent manner, together driving CC cell proliferation [288]. Among circRNAs, circARHGAP12 interacts with IGF2BP2 through m^6^A to enhance FOXM1 mRNA stability. Moreover, circCCDC134 maintains its own stability through ALKBH5-regulated m^6^A and promotes metastasis by recruiting p65 to increase the transcription of hypoxia-inducible factor 1-alpha (HIF1α) [87,89]. In addition, Piwi-interacting RNA (piRNA)-14633 can increase METTL14 expression, elevating cytochrome P450 family 1 subfamily B member 1 (CYP1B1) mRNA and protein levels and promoting malignant phenotypes in CC cells [94].

From a translational perspective, m^6^A regulators show promise for a diagnosis, prognosis and treatment response assessment in CC. Prognostic models based on m^6^A-related genes such as ZC3H13 and YTHDC1 can help predict patient survival. The evaluation of combinations of METTL3 MDSCs and CD33-positive myeloid-derived suppressor cells (MDSCs) will be more helpful [297,298]. STM2457, an METTL3 inhibitor, and CWI1-2, an IGF2BP2 inhibitor, show antitumor effects in acute myeloid leukaemia. Rhein, an anthraquinone compound, has the potential to treat breast cancer. Further studies should investigate whether these agents can act on CC and other tumour types [299,300,301]. In addition, the findings in gastric cancer and leukaemia imply that the m^6^A-targeted therapies mediated by nanocarriers may be highly specific and less toxic, given the pro-tumour function of YTHDF1 in CC. This strategy could indicate a possible route for precision therapy in CC [302,303].

Recent investigations have initiated the mapping of the RNA modification landscape in CC. Writers such as METTL3 and METTL14 for m^6^A have been implicated in tumorigenesis via the stabilisation of the HPV E6 and E7 mRNAs, while ALKBH5 and FTO have been reported to be tumour suppressive in some settings.

Other than m^6^A, the m^5^C NSUN2 culprit and the m1A TRMT6 and TRMT61A culprits have also been implicated in cervical cancer cell models. However, there are many limitations. Numerous studies have focused on a single enzyme and a linear downstream target, while the potential competition or cooperation of marks, as shown by m^6^A and m^5^C, is often overlooked. Whether HPV16-positive and HPV18-positive tumours display distinct epitranscriptomic features also remains insufficiently compared. A significant portion of existing research relies heavily on in vitro models (e.g., SiHa, HeLa or SKOV3 cell lines) and high-dose knockdown/overexpression experiments, which may not faithfully recapitulate the physiological complexity of the human reproductive microenvironment. The therapeutic potential of combining small-molecule inhibitors targeting FTO or METTL3 with PD-1 and PD-L1 antibodies or platinum-based chemotherapy also requires validation. Future studies should integrate CLIP-seq and RNA-seq to map interactions among RNA modifications and use patient-derived organoids and HPV molecular subtyping cohorts to clarify tumours’ heterogeneity. Developing cervical cancer-specific inhibitors of RNA modification enzymes and testing strategies that combine these agents with tumour microenvironment remodelling may offer new approaches to overcome the resistance to chemoradiotherapy.

### 4.2. Ovarian Cancer

Within the m^6^A methyltransferase complex, METTL3 mainly works as a cancer-promoting factor in ovarian cancer. METTL3 is highly expressed in ovarian cancer tissue and is tightly linked to a higher tumour grade. Hua et al. demonstrated that METTL3 promotes ovarian cancer growth and invasion by increasing the translation of AXL receptor tyrosine kinase and facilitating EMT [304]. METTL3 can also enhance tumour cell survival and proliferation by activating the phosphoinositide 3-kinase/protein kinase B/mechanistic target of rapamycin (PI3K/AKT/mTOR) signalling. In ovarian cancer, METTL3 can promote the maturation of miR-126-5p in an m^6^A-dependent manner, which suppresses PTEN and activates this pathway. METTL3 may also promote progression by regulating AKT phosphorylation and downstream effectors [68,305]. In endometrioid epithelial ovarian cancer, METTL3 can work without METTL14 and WTAP and can regulate m^6^A on genes such as EIF3C and AXL [306]. Conversely, METTL14 is usually considered tumour suppressive in ovarian cancer. METTL14 expression is reduced in ovarian cancer tissue, and METTL14 can inhibit G1-phase proliferation by decreasing the stability of TROAP mRNA [307]. WTAP is the main regulatory subunit of the m^6^A writer complex. Although WTAP lacks catalytic activity, it helps maintain complex functioning by directing METTL3 and METTL14 to nuclear speckles. Yu et al. reported that WTAP is markedly elevated in high-grade serous ovarian carcinoma (HGSOC) and that higher WTAP levels are tied to lymph node metastasis and shorter overall survival. In vitro, WTAP knockdown suppresses proliferation and migration and induces apoptosis, indicating the cancer-promoting function of WTAP in ovarian cancer progression [308].

Moreover, m^6^A readers have key functions in ovarian cancer progression. In cisplatin-resistant ovarian cancer cells, increased m^6^A on TRIM29 mRNA recruits YTHDF1 and enhances translation, maintaining stem-like features [308]. YTHDF1 can also bind EIF3C mRNA and increase its translation, supporting the progression of ovarian cancer [309]. YTHDF2 forms a double-negative feedback loop with miR-145 and promotes proliferation and migration by reducing global m^6^A levels [310]. The E3 ubiquitin ligase FBW7 (F-box and WD repeat domain-containing 7) can counter this effect by inducing the proteasomal degradation of YTHDF2 and restricting its cancer-promoting effects [311]. In addition, IGF2BP1 works as an m^6^A reader that can stabilise SRF mRNA to promote ovarian cancer progression or stabilise MDM2 mRNA to facilitate immune evasion and tumour growth [312,313].

The m^6^A demethylase FTO appears to be tumour suppressive in high-grade serous ovarian carcinoma. Huang et al. found that FTO reduces m^6^A and the stability of PDE1C and PDE4B mRNAs, strengthens cyclic adenosine monophosphate (cAMP) signalling and suppresses the self-renewal of ovarian cancer stem cells. In contrast to its pro-tumour roles reported in leukaemia and glioblastoma, these findings show that FTO functions vary by tissue [314]. Conversely, ALKBH5 mainly shows pro-tumour activity in ovarian cancer. Jiang et al. reported that ALKBH5 is highly expressed and is regulated by the toll-like receptor 4 (TLR4) and NF-κB signalling axis in the tumour’s microenvironment. Through m^6^A demethylation, ALKBH5 increases NANOG expression and promotes tumorigenesis [315]. ALKBH5 can also form a positive feedback loop with HOXA10 and drive cisplatin resistance by demethylating Janus kinase 2 (JAK2) mRNA [316].

M^6^A is also involved in shaping the immune microenvironment and chemotherapy-related phenotypes in ovarian cancer. Such m^6^A regulators as ZC3H13, YTHDF1 and IGF2BP1 are tied to tumour immune infiltration [317]. In addition, elevated m^6^A on FZD10 mRNA has been linked to resistance to poly (ADP-ribose) polymerase (PARP) inhibitors by increasing transcript stability and activating Wnt and β-catenin signalling [318].

Bioinformatic analyses using public resources, such as TCGA and GEO, indicate that the dysregulated expression of m^6^A regulators is tightly linked to ovarian cancer prognoses. Fan et al. used LASSO Cox regression to identify IGF2BP1, VIRMA and ZC3H13 for a risk score model. Higher expression was tied to worse outcomes, and these genes were enriched in WNT signalling and cancer-related pathways [319]. Zhu et al. broadened prognostic models by including KIAA1429, YTHDC2, HNRNPC and WTAP and noted that the resulting risk score predicted survival regardless of age and tumour stage. In this model, a higher expression of KIAA [320].

Other RNA marks outside m^6^A also promote ovarian cancer’s progression. In platinum resistance, m^5^C regulates the NSUN2 ALYREF LGR4 axis that enhances the tolerance to cisplatin. NSUN2 adds m^5^C to the coding region of leucine-rich repeat-containing G-protein coupled receptor 4 (LGR4) mRNA, a leucine-rich repeat-containing G-protein coupled receptor that is recognised by ALYREF. This interaction increases transcript stability and supports nuclear export, leading to the activation of Wnt and β-catenin signalling and, finally, the development of a platinum-resistant phenotype [321].

Additionally, m7G plays a role in ovarian cancer. METTL1 has been reported to increase BRCA1 expression by installing m7G on breast cancer susceptibility gene 1 (BRCA1) mRNA and promoting proliferation and tumour growth in high-grade serous ovarian cancer. A vaginal microbiota-derived metabolite, 5-formamidoimidazole-4-carboxamide ribotide (FAICAR), can bind METTL1 and inhibit its methyltransferase activity, reduce m7G on BRCA1 and suppress tumour progression. These findings suggest that host microbe interactions can influence ovarian cancer through epitranscriptomic regulation [322]. In the regulation of stem-like traits, the ac4C acetyltransferase NAT10 increases the stability of cytoplasmic activation/proliferation-associated protein 1 (CAPRIN1) mRNA through ac4C and thus promotes ovarian cancer cell migration, invasion and maintenance of stem-like properties [323]. Currently, the evidence linking METTL3 to ovarian cancer progression is robust across multiple independent cohorts, whereas the roles of rarer modifications such as ac4C and m7G in reproductive health remain in the exploratory phase, primarily supported by descriptive sequencing data rather than mechanistic validation [134,304].

### 4.3. Endometrial Cancer

The aberrant regulation of RNA methylation is an important molecular mechanism in endometrial cancer (EC). That is, m^6^A and its regulators show changed expression patterns in EC tissue and drive tumour proliferation, invasion and metastasis by affecting mRNA stability, translation and signalling pathway activity. Low METTL3 expression or functional defects in METTL14 are common in EC. Reduced m^6^A can enhance AKT signalling by decreasing the AKT-negative regulator PHLPP2 and increasing the positive regulator mTORC2, promoting EC cell proliferation and tumorigenicity [324]. Conversely, WTAP is highly expressed in EC and promotes progression by installing m^6^A within the three prime untranslated regions of Caveolin-1 (CAV-1) mRNA and reducing its expression, which activates NF-κB signalling [325].

M^6^A readers have dual effects on EC. YTHDF2 works as a tumour suppressor and can bind m^6^A-marked insulin receptor substrate 1 (IRS1) mRNA and promote its decay, limiting IRS1 and AKT signalling [326]. Conversely, IGF2BP1 works as an oncogene and is highly expressed in EC. IGF2BP1 recognises m^6^A sites in the three prime untranslated regions of Paternally Expressed Gene 10 (PEG10) mRNA and recruits Polyadenylate-binding protein 1 (PABPC1) to increase PEG10 mRNA stability, which promotes EC cell proliferation. Higher IGF2BP1 expression is also tied to worse outcomes [327]. Reduced m^6^A levels may further promote EC proliferation and tumour formation through a pregnancy-associated plasma protein-A (PAPPA) and insulin-like growth factor binding protein 4 (IGFBP4) axis that modulates extracellular signal-regulated kinase (ERK) along with NF-κB and AKT signalling [328].

Among erasers and related enzymes, both FTO and ALKBH5 drive EC progression. FTO limits the decay of Homeobox B13 (HOXB13) mRNA through demethylation, increases HOXB13 protein levels and activates Wnt signalling to enhance invasion and metastasis [329]. ALKBH5 increases the stability and translation of insulin-like growth factor 1 receptor (IGF1R) mRNA through demethylation and promotes EC cell proliferation [329]. Under hypoxia, ALKBH5 can also maintain the expression of SRY-box transcription factor 2 (SOX2) mRNA and strengthen stem-like phenotypes in EC [330]. In addition, Peptidylarginine Deiminase 2 (PADI2) can activate ERK1 and ERK2 signalling through MEK1 citrullination and enhance the IGF2BP1-mediated stabilisation of SOX2 mRNA. This PADI2 MEK1 ERK IGF2BP1 SOX2 axis may drive malignant progression in EC [331].

Prognostic models based on m^6^A regulator expression could aid in EC risk assessment. Low FTO expression and high RBM15 expression have been tied to poor prognoses and a higher FIGO grade in endometrial adenocarcinoma. A risk score derived from FTO, RBM15 and YTHDF1 can predict survival outcomes. Various target genes, including IGF1 and IRS1, can influence energy metabolism, RNA stability and connective tissue formation. The progression of this malignancy may also be influenced by FTO through its effects on EMT and chemotherapy sensitivity, highlighting the possibility that targeting m^6^A enzymes and associated pathways may be useful for the design of precision treatment strategies in endometrial adenocarcinoma [332].

RNA modifications beyond m^6^A also promote EC progression. Post-transcriptional modifications such as m^5^C, m^1^A and m^7^G can modulate the stability and function of lncRNAs or messenger RNAs. The accumulation of such modifications can assist with the remodelling of the immune microenvironment, control of the cell cycle and response to treatment in EC. A risk model based on m^5^C-related lncRNAs correlates with PD-L1 expression and immune cell infiltration and can predict patient outcomes [333]. Patterns associated with m1A have been linked to immune pathway enrichment and tumour mutational burden and can distinguish between immune-inflamed and immune-cold phenotypes [147]. M7G is a type of cap found on the messenger RNA molecule of eukaryotic cells. Such m7G-related mRNAs have been connected to tumours’ progression through effects on stemlike properties, immune infiltration and drug sensitivity [334]. Collectively, our findings reveal that multiple RNA modifications collaborate to regulate EC development and progression and that these findings may identify new targets for patient stratification and precision therapy.

### 4.4. Choriocarcinoma

Choriocarcinoma is a highly invasive tumour of trophoblastic origin that invades blood vessels early and metastasises widely. The tumour that arises due to the uncontrolled multiplication of cytotrophoblasts and syncytiotrophoblasts can be gestational or non-gestational. The gestational type tends to be relatively chemosensitive; however, nearly 30% show systemic metastasis on the first presentation and 5% display multidrug resistance at presentation. The form that is not gestational has a more aggressive course: 84% of patients eventually die from the disease despite multiagent chemotherapy, indicating broad resistance to therapy. Distinguishing these two origins remains a clinical challenge that can affect the choice of therapy and prognosis assessment [335,336]. Recent findings suggest that m^6^A likely plays a role in the progression of choriocarcinoma. This modulation may occur through an ncRNA network, though the associated mechanistic details remain elusive.

METTL3, the m^6^A writer, was identified as a critical regulator of choriocarcinoma progression. METTL3 plays a role in processing pri-miR-935, which reduces the expression of gap junction protein GJA1 (Cx43). This suppression weakens intercellular communication and enhances the growth and invasion of choriocarcinoma cells [93]. METTL3 enhances the maturation of pri-miR-21 and boosts miR-21-5p levels. The latter targets the 3′-untranslated region of hypoxia-inducible factor asparagine hydroxylase (HIF1AN) mRNA, enhancing its decay. The reduced expression of HIF1AN relieves the inhibition of HIF1A, enhancing both HIF1A activity and vascular endothelial growth factor (VEGF) expression. Optimised angiogenesis through this pathway supports the growth and spread of tumours [337].

Some ncRNAs play tumour-suppressive roles in choriocarcinoma. The upregulation of miR-373-3p in cancer inhibits invasion and metastasis by targeting transforming growth factor β receptor II (TGFβR2), reducing TGFβ signalling and the epithelial–mesenchymal transition [338]. According to these observations, m^6^A regulatory networks exert context-dependent bidirectional effects in choriocarcinoma on distinct RNA substrates and cell contexts.

Besides ncRNA modulation, m^6^A directly modulates the stability and expression of protein-coding transcripts. In an m^6^A-dependent manner, phospholipase Cε1 (PLCE1) mRNA is stabilised by METTL14. Subsequently, PLCE1 induces VEGF secretion with Ras-associated protein 1 (Rap1) signalling, which promotes proliferation, the epithelial–mesenchymal transition and angiogenesis [339]. Previously, METTL3 was shown to interact with YTHDF3 to deposit m^6^A onto phosphoglycerate kinase 1 (PGK1) mRNA, thereby enhancing both transcript stability and translational efficiency to drive glycolytic reprogramming and malignant progression [340].

There is still insufficient knowledge about how epitranscriptomics influence the disease. Thus far, research has focused on m^6^A, especially writers (METTL3 and METTL14) and readers (YTHDF3). The systematic characterisation of demethylases, including FTO and ALKBH5, along with other alternative YTH family members, has yet to be achieved. CircRNA and lncRNA are excellent candidates for other marks, such as m^5^C and A-to-I RNA editing, and other modifications that remain to be deciphered. Broadening the research scope beyond m^6^A-centric paradigms for the construction of the interactomes of multiple RNA modifications will enable a better understanding of epitranscriptomic governance in choriocarcinoma and facilitate the development of precision therapeutics.

While the oncogenic roles of RNA modifications in gynaecologic malignancies are well established, their influence extends to a broader range of reproductive health issues. The precise spatio–temporal regulation of the epitranscriptome is equally critical for the maintenance of pregnancy. Disruption of these finely tuned mechanisms can lead to a variety of pregnancy-related disorders, including preeclampsia, gestational diabetes mellitus and miscarriage, as discussed below.

## 5. RNA Modification Regulation in Pregnancy-Related Disorders

### 5.1. Preeclampsia

Preeclampsia (PE) is a pregnancy disorder characterised by fresh hypertension, along with proteinuria or end-organ dysfunction. Approximately 2% to 5% of pregnancies worldwide are affected by this condition. It is a leading cause of maternal and perinatal mortality [341]. The inadequate trophoblast invasion at an early stage and the failure to remodel the uterine spiral arteries are believed to be the underlying causes of pathogenesis, leading to ischemia and hypoxia of the placenta. These insults lead to the haemodynamic release of antiangiogenic factors and proinflammatory mediators, ultimately resulting in maternal endothelial injury, systemic inflammation and multi-organ dysfunction [342]. At present, screenings and prevention strategies are limited. Low-dose aspirin confers advantages only to certain high-risk groups, and delivery is the sole curative intervention. In addition, affected pregnancies bear a notably higher long-term cardiovascular risk [343]. Deciphering epigenetic regulatory mechanisms, such as RNA modifications, represents a major goal for understanding disease pathophysiology and forging new therapeutic interventions.

Analyses of epitranscriptomic patterns reveals heterogenous m^6^A in PE-induced placentas. Taniguchi et al. linked m^6^A deposition within placental mRNAs’ 5′-untranslated region (5′UTR) to foetal growth restriction and PE [264]. Zhu et al. reported increased levels of m^6^A and METTL3 expression in trophoblast cells from the placenta of PE [344]. Conversely, Bian et al. demonstrated that via an IGF2BP1-HMGN3 axis, reduced m^6^A could block trophoblast invasion [345]. This variation likely mirrors the heterogeneity underlying PE pathogenesis. However, some studies focusing on biomarkers are limited by small cohort sizes and a lack of multicentre validation, which weakens the statistical power and clinical generalisability of the findings [346].

The activity of m^6^A writers and readers modifies trophoblast behaviour via specific target transcripts. METTL3 promotes trophoblast invasion by stabilising myosin light chain kinase (MYLK) mRNA; disruption of this mechanism is associated with PE [266]. The activity of m^6^A writers and readers modifies trophoblast behaviour via specific target transcripts. METTL3 promotes trophoblast invasion by stabilising myosin light chain kinase (MYLK) mRNA; disruption of this mechanism is associated with PE [347]. Further studies depict that METTL3 stabilises acyl-CoA synthetase long-chain family member 4 (ACSL4) mRNA and promotes trophoblast ferroptosis, thus accelerating PE development [348]. The uplifted expression of METTL14 is also associated with the dysfunction of trophoblasts through m^6^A deposition on forkhead box O3a (FOXO3a) mRNA and the increased stability of transcripts [349]. The overexpression of RBM15 enhances the binding of YTHDF2 to CD82 mRNA, thereby speeding up its degradation and reducing the expression of CD82 mRNA [350].

According to the impact of the writer and the reader, an m^6^A eraser possesses dual regulatory abilities in PE. PE placentas demonstrate increased levels of ALKBH5, which may worsen the disease by reducing the stability of the peroxisome proliferator-activated receptor γ (PPARγ) mRNA [269]. Nevertheless, ALKBH5 enhances the expression of placenta-specific 8 (PLAC8) by eliminating m^6^A from PLAC8 mRNA, thus facilitating trophoblast invasion and migration, which may be advantageous in PE [351]. ALKBH5 confers a context-dependent function, so the directionality of activity is determined by the specific cell type and cell-type-specific downstream targets.

Beyond mRNAs, m^6^A regulates the function of non-coding RNAs. Wang et al. uncovered that the maintenance of the stability of the HOXD cofactor cluster antisense RNA 1 (HOXD-AS1) was influenced by METTL3-mediated m^6^A modification on long non-coding RNAs. HOXD-AS1 sequesters cellular microRNA miR-135a and promotes the expression of β-transducin repeat-containing protein (β-TRCP) and NF-κB signalling to modulate PE progression [352]. Zhang et al. discovered that the m^6^A manipulation of linc01116 takes place with the help of IGF2BP2. Linc01116, which is lessened in PE placentas, sponges miR-210-3p to promote angio-associated migratory cell protein (AAMP) expression, which promotes trophoblast angiogenesis and invasion [353]. A circSETD2-miR-181a-5p-myeloid cell leukaemia 1 (MCL1) axis represents another regulatory node regarding circRNA regulation. The METTL3 m^6^A-mediated stabilisation of circSETD2 functions as a competing endogenous RNA that binds to miR-181a-5p, relieving MCL1 suppression and alleviating trophoblast apoptosis [354]. Likewise, in placentas with PE, m^6^A mediated by METTL14 on circPAPPA2 increases, with the stability of circPAPPA2 being sustained by IGF2BP3 in an m^6^A-dependent manner. PE loss hampers circPAPPA2 and alters trophoblast invasion via IGF2BP3 expression [355].

Family members NOP2/Sun RNA methyltransferase and m^5^C participate in PE pathology. Enoxaparin enhances trophoblast functioning by activating NSUN2-mediated m^5^C and stabilising the mRNA of paired box 3 (PAX3) [356]. NSUN5 is associated with decidualisation by regulating the cellular energy metabolism through its interaction with ATP synthase F1 subunit beta (ATP5B) [357]. An R295C variant at rs77133388 in NSUN5 dampens IL-11Rα-JAK2-STAT3-Cyclin D3 signalling, impairs decidual cell polyploidisation and thereby increases the risk for PE [358]. The m^5^C regulatory network contributes to the pathogenesis of PE. Similarly to PE, other common pregnancy complications, such as gestational diabetes mellitus, exhibit profound alterations in the epitranscriptomic landscape, highlighting the broad relevance of RNA modifications in gestational health.

### 5.2. Gestational Diabetes Mellitus

Gestational diabetes mellitus (GDM) is the most common metabolic complication in pregnancy. This condition occurs when there is worsening physiological insulin resistance during pregnancy, together with insufficient compensatory ability of pancreatic β-cells [359]. Despite the widespread acceptance of the diagnostic criteria laid down by IADPSG and WHO 2013, considerable heterogeneity in prevalence estimates is produced by regional differences in screening strategies. GDM increases the risk of hypertensive disorders during pregnancy and foetal overgrowth in the mother. It also increases both the mother’s and child’s risk for metabolic disease through in utero metabolic programming. Understanding the molecular mechanisms of GDM, especially epigenetic RNA modification leading to glucose homeostasis, will help ameliorate diagnosis and treatment [360].

The expression of METTL14, a component of the m6A methyltransferase complex, is altered in GDM. Placental tissue from patients with GDM has lower METTL14 expression and m6A deposition on BMP- and activin-membrane-bound inhibitor (BAMBI) mRNA and BAMBI expression. Alterations in these pathways may modulate transforming growth factor-β (TGF-β) and Wnt signalling, impair β-cell function and promote insulin resistance, which raises the risk of GDM [361]. METTL14 in GDM pathogenesis through the XIST-miR-497-5p-FOXO1 axis. In high-glucose-cultured HTR-8/SVneo cells, METTL14 mediates the m^6^A-dependent silencing of X-inactive specific transcript (XIST) in high-glucose cultured HTR-8/SV neo cells. The silencing of this gene decreases the sequestration of microRNA-497-5p (miR-497-5p) by XIST and increases miR-497-5p levels, downregulating forkhead box O1 (FOXO1). These modifications increase the proliferation and migration of trophoblasts while reducing apoptosis, which may inhibit GDM development [362]. M^6^A on chemokine ligand 5 (CCL5) by METTL14 controls CCL5 mRNA stability and influences the proliferation, migration and apoptosis of trophoblasts, thereby altering GDM progression [363].

Outside METTL14, METTL3 is active in the regulation of GDM. The M^6^A methylation of hsa_circ_0072380 by METTL3 reduces circular RNA stability and may affect the GDM mechanism by altering trophoblast proliferation, migration and invasion [364]. Regarding m^6^A demethylases, FTO demethylates the mRNA of salt-inducible kinase 1 (SIK1), ultimately reducing its stability and expression. Thus, cytotrophoblast fusion is inhibited, leading to defective placental syncytialisation [365]. Concerning m^6^A demethylases, FTO demethylates the mRNA of salt-inducible kinase 1 (SIK1), ultimately reducing its stability and expression. Thus, cytotrophoblast fusion is inhibited, leading to defective placental syncytialisation [366]. RBM15 regulates the m^6^A level of claudin 4 (CLDN4) gene expression and plays a role in the glucose and lipid metabolism in the offspring of GDM mice. It might cause insulin resistance [367]. Hesperidin is a therapeutic, natural compound of potential. Trophoblast cells from obese GDM patients have increased m^6^A levels; hesperidin exerts protective effects through autophagy and m^6^A methylation [368].

The complicated and rich world of idolatries advances as we enter the world of idolatries. Under high-glucose conditions, NSUN2 modifies PTEN-induced putative kinase 1 (PINK1) mRNA and alters mitophagy, events that impair trophoblast functioning. The inhibition of NSUN2 enhances the glucose metabolism and decreases inflammation in pregnant GDM mouse models [369]. Furthermore, GDM placenta tissue accumulates n4-acetylcytidine. N-acetyltransferase 10 (NAT10) alters mesenchyme homeobox 2 (MEOX2) mRNA while regulating human umbilical vein endothelial cell (HUVEC) angiogenesis. NAT10 inhibition alleviates the endothelial dysfunction induced by high glucose concentrations [370].

RNA modifications operate as fundamental modulators of GDM, functioning across trophoblast functions, placental angiogenesis and insulin sensitivity through marks including m^6^A, m^5^C and ac4C. Nevertheless, much evidence is derived from in vitro cell systems and animal models, while crosstalk among modification enzymes and pregnancy-stage-specific modification dynamics awaits elucidation. Prospective investigations warrant the integration of multi-omics approaches to clarify GDM-specific interactions among RNA modifications and the development of targeted interventions against key enzymes capable of acting across the placental barrier. Such advances may help curtail the pathological transmission of in utero metabolic programming and improve long-term outcomes for both mother and child.

### 5.3. Miscarriage

Miscarriage denotes involuntary pregnancy loss before 24 weeks of gestation, affecting approximately 23 million cases globally each year (44 cases per minute), representing over 15% of all clinically confirmed pregnancies. Recurrent spontaneous abortion (RSA) traditionally refers to the loss of three or more consecutive pregnancies; however, the European Society of Human Reproduction and Embryology revised this definition in 2018 to encompass two or more non-consecutive pregnancy losses, a condition affecting roughly 1–2% of women of reproductive age [371,372,373]. Although chromosomal abnormalities, endocrine disorders and anatomical factors constitute established aetiologies, approximately half of recurrent miscarriages are unexplained. RNA modifications in embryo development and pregnancy maintenance have drawn growing research interest, offering new insights into molecular bases of idiopathic pregnancy loss.

The expression of METTL3 is reduced in placental villi from patients with RSA. The m^6^A modifications induced by METTL3 generally destabilise Zinc Finger and BTB domain containing 4 (ZBTB4) mRNA and inhibit its expression. Decreased METTL3 activity allows the accumulation of ZBTB4, which decreases the proliferation and migration of trophoblasts [374]. Conversely, environmental pollutants such as benzo(a)pyrene (BaP) and its metabolite benzo(a)pyrene-7,8-dihydrodiol-9,10-epoxide (BPDE) upregulate METTL3 expression, increasing the m^6^A levels of lncRNA lnc-HZ09 and enhancing stability. This change inhibits the phospholipase d1 (PLD1)/RAC family small GTPase 1 (RAC1)/cell division cycle 42 (CDC42) signalling axis, reducing trophoblast migration and invasion and leading to RSA [375]. Exposure to BPDE damages pyroptosis via the lnc-HZ14/Z-DNA binding protein 1 (ZBP1)/NLRP3 axis. ZBP1 transcription is promoted by lnc-HZ14 through interferon regulatory factor 1 (IRF1). It is also involved in NLRP3 mRNA m^6^A modification and stabilisation through METTL3, which leads to pregnancy loss [376].

At the same time, METTL14 expression increased in human trophoblasts and villous tissue from recurrent miscarriage patients exposed to BPDE. The METTL14 gene leads to increased lnc-HZ01’s m^6^A modification and enhanced RNA stability. This process creates a feedback loop involving MXD1, METTL14 and lnc-HZ01. The abnormal triggering of this loop significantly inhibits the growth of trophoblasts. Mechanistically, lnc-HZ01 upregulates transcription factor c-JUN and deubiquitinating enzyme ubiquitin-specific protease 36 (USP36), which increases the mRNA transcription and protein stability of MAX dimerisation protein 1 (MXD1). Later, MXD1 activates lnc-HZ01 expression by inducing METTL14 transcription to catalyse m^6^A methylation [377].

The literature presents paradoxical findings regarding the role of the m^6^A demethylase ALKBH5 in RSA, highlighting a critical need for the rigorous appraisal of the experimental models. ALKBH5 is notably upregulated in placental villous tissue from RSA patients, leading to a global reduction in m^6^A levels. This downregulation shortens the half-life of cysteine-rich protein 61 (CYR61) mRNA, disrupting trophoblast invasion while reducing VEGF secretion, which impairs macrophage recruitment and M2 differentiation at the maternal–foetal interface [377]. Conversely, Zheng et al. demonstrated that ALKBH5 expression was markedly decreased in the trophoblasts of RSA patients. In their model, hypoxia induces the nucleocytoplasmic translocation and enrichment of ALKBH5, which removes m^6^A marks to enhance the expression of matrix metalloproteinase 9 (MMP9) and integrin α1 (ITGA1) via the SMAD1/5 signalling axis, promoting trophoblast viability and invasion [378]. The varying findings stem from cell-type specificity and oxygen–environment dependence. ALKBH5 acts through the VEGF and SMAD1/5 pathways in uterine stromal cells and extravillous trophoblasts. Moreover, hypoxia-inducible factor 1-alpha (HIF-1α) signalling is activated by different oxygen tensions, modulating early pregnancy trophoblast activity. The specificity and environmental dependence of target gene selection suggest that ALKBH5 is context dependent and may modulate pregnancy maintenance in opposite ways. First, the spatio–temporal heterogeneity of placental development means that ALKBH5 activity may vary significantly between the first and second trimesters. Second, the methodological differences in m6A quantification (e.g., MeRIP-seq vs. LC-MS/MS) and the diverse cell types (primary villi vs. immortalised cell lines such as HTR-8/SVneo) utilised across studies likely contribute to these inconsistent findings. Therefore, future researchers must employ single-cell resolutions and standardised functional assays to resolve these contradictions and clarify whether ALKBH5 serves as a protective or detrimental factor in the maternal–foetal interface [162].

The downregulation of another m^6^A demethylase, FTO, occurs in the maternal–foetal interface of spontaneous abortion patients. FTO deficiency leads to abnormal m^6^A build-up in villous tissue and oxidative stress, which downregulates human leukocyte antigen-G (HLA-G) and VEGFR expression while upregulating matrix metalloproteinases (MMP) 7 and MMP9. These changes affect maternal–foetal immune tolerance and angiogenesis, thus causing a miscarriage [379].

The m7G modification in unexplained recurrent spontaneous abortions or unexplained recurrent spontaneous abortions (URSA) is being researched. Transcriptomic analyses of decidua depicted the upregulation of Sm-like protein 1 (LSM1) with the downregulation of La-Related Protein 1 (LARP1) and Nuclear Cap-Binding Protein Subunit 2 (NCBP2) in URSA patient decidua. Possible diagnostic biomarkers could be these three proteins. Furthermore, URSA patients displayed a low density of decidual regulatory T cells (Tregs) and a high density of helper T cells (Th). Notably, LSM1 was negatively correlated with the infiltration of Tregs, while LARP1 and NCBP2 exhibited a positive correlation with Tregs [380]. According to these observations, m7G modification helps regulate Treg homeostasis and the maternal–foetal immune tolerance imbalance. This offers a new epitranscriptomic perspective in understanding the mechanisms of immune dysregulation in URSA, which indicates that m7G regulatory factors may be targeted for precise diagnosis and therapy in URSA.

RNA modifications also play a pivotal role in the pathogenesis of chronic reproductive endocrine disorders, such as PCOS and premature ovarian insufficiency. While pregnancy disorders often involve acute placental or immune dysregulation, endocrine disorders typically reflect long-term disturbances in ovarian and metabolic homeostasis, both underpinned by complex epitranscriptomic networks (Table 2).

## 6. Regulation of RNA Modifications in Reproductive Endocrine Disorders

### 6.1. Polycystic Ovary Syndrome

PCOS is the most common endocrinological and metabolic disorder among women of reproductive age and affects nearly 10% of the world’s women. This condition is associated with hyperandrogenism, ovulatory dysfunction and polycystic ovarian morphology. PCOS is a genetic predisposition and metabolic disorder affected by the environment. The core pathophysiological mechanisms, that is, insulin resistance (IR), compensatory hyperinsulinemia and adipose tissue dysfunctions, set up a vicious cycle between the metabolic and reproductive axes [394,395]. With RNA modifications being important molecular mechanisms that affect epigenetic regulation in metabolic homeostasis and germ cell development, understanding their functional roles in PCOS pathophysiology could unravel molecular aetiology and suggest targeted interventions.

In a meta-analysis of 2548 PCOS patients, Wojciechowski et al. found that the fat mass and obesity associated gene (FTO) rs9939609 variant has almost double the genetic effect on body mass index (BMI) and body weight in PCOS compared to the general female population. This observation indicates that the different metabolic environments or genetic backgrounds of PCOS influence FTO variations in obesity-related traits. This study mainly included European white populations and may not be applicable to Asian groups [396]. A separate study of Chinese women with a PCOS diagnosis found no significant independent association of FTO variation with the obesity phenotype, suggesting the effect is ethnic specific [397]. The granulosa cell (GC) expression of FTO in patients with PCOS is increased, showing a negative correlation with intracellular m^6^A levels and a positive correlation with follicular fluid androgen concentrations. Through its mechanism of action, androgen receptor (AR) activation triggers increased FTO levels. Subsequently, FTO enhances steroid hormone biosynthesis, which worsens hyperandrogenism while creating a positive feedback loop [226]. In another context, FTO reduces the m^6^A modification of the flotillin 2 (FLOT2) mRNA and enhances its stability, promoting GC proliferation, etc. This FTO/FLOT2 axis is considered crucial in the pathological development of PCOS [227].

Apart from FTO, the altered expression of IGF2BP2 leads to GC dysfunctioning in PCOS. The expression of IGF2BP2 is upregulated in the GCs of PCOS patients and promotes cell proliferation via the mRNA binding and stabilisation of cyclin D2 (CCND2) and SERPINE1 mRNA binding protein 1 (SERBP1) mRNAs [398]. In PCOS patients, GCs exhibit increased expression of YTHDF2. The translation-activating protein for mitochondrial mss51 mRNA of this reader is recognised, bound and downregulated, impairing mitochondrial function and glycolysis and causing GC dysfunction [384].

Intestinal microbe dysbiosis is closely associated with the m^6^A epitranscriptomic abnormalities of PCOS. Investigations have demonstrated that obese PCOS patients exhibit aberrant gut microbiota diversity accompanied by reduced serum butyrate levels. Butyrate supplementation inhibits the expression of METTL3, reduces m^6^A modification on FOS-like antigen 2 (FOSL2) mRNA and inhibits NLRP3 inflammasome activation and inflammatory cytokines, such as IL-6 and TNF-α, which improves ovarian functioning and inflammatory status [227]. In luteinised GCs from non-obese PCOS patients, for example, FOXO3 mRNA, m^6^A modification levels are significantly downregulated, which boosts FOXO3 mRNAs’ stability and YTHDF2-mediated RNA degradation and expression and influences apoptosis metabolism and proliferation [228].

A PCOS patient may be phenotypically obese or lean, insulin resistant or noninsulin resistant. The precise regulatory schemes of m^6^A modifications among subtypes have yet to be defined [67]. The m^6^A-modifying enzymes and reader proteins have the potential of being useful diagnostic biomarkers and therapeutic targets for PCOS. However, large-cohort studies are needed to confirm the molecular relationships of genetic variations in FTO with phenotypes such as ovulatory dysfunction and the regulatory roles of dietary habits and metabolic states on m^6^A modifications in women with PCOS [61].

NAT10-mediated ac4C modification also regulates PCOS. PCOS patient ovarian tissues exhibit significantly reduced NAT10 expression and ac4C levels. NAT10 restricts abnormal GC proliferation by enhancing the mRNA stability of C-X-C motif chemokine ligand 14 (CXCL14). Animal experiments have established that localised ovarian NAT10 overexpression ameliorates cystic follicle formation and hyperandrogenism via the NAT10/CXCL14 axis [399]. Simultaneously, GC pyroptosis in PCOS is controlled by NSUN7. Its expression is elevated in PCOS patients, and this enzyme stabilises the mRNA of NLRP3 through m^5^C catalysis and promotes the assembly of the NLRP3 inflammasome and cellular pyroptosis. The inhibition of NSUN7 ameliorates hormonal imbalances and the pathology of the ovary, suggesting that this axis is a potential target for PCOS [231]. Furthermore, significant A-to-I RNA editing alterations occur in the GCs of PCOS patients. Transcriptome analysis identified 545 differential editing sites that are significantly enriched in genes nucleoporin 43 (NUP43) and retinoblastoma binding protein 4 (RBBP4), which are involved in apoptosis and necroptosis. The downregulation of ADARB1, an RNA editing enzyme, in the PCOS condition might indicate that A-to-I editing might influence PCOS development by altering the expression of GC survival [106]. This study offers significant insights into the regulatory mechanism of RNA modifications in PCOS. It will help develop disease-specific therapeutics and add to the understanding of the molecular heterogeneity of the condition.

### 6.2. Premature Ovarian Insufficiency

Premature ovarian insufficiency (POI), defined as ovarian failure before the age of 40, presents as amenorrhea and elevated FSH. Its global prevalence approximates 1–3.5%. However, this figure shows significant ethnic and geographic variations. The depletion of the primordial follicle pool is accelerated, the premature atresia of follicles occurs, and meiotic regulation is disturbed. Infertility and long-term complications such as osteoporosis, cardiovascular disease and neurocognitive impairment are associated with these processes [400,401]. While investigations of FMR1 premutation, genetic defects in meiosis and the like targeted variations at the DNA sequence level, more recent evidence points to epitranscriptomic regulation, particularly RNA modifications, to maintain follicular homeostasis by regulating mRNA stability and translational efficiency, as well as the metabolic reprogramming of oogenesis-associated genes. Understanding the RNA modification regulatory networks involved in the pathogenesis of POI is important for revealing the molecular mechanism of ovarian ageing, further screening of early diagnostic biomarkers and developing precise intervention strategies for POI.

The genetic mechanisms linked to POI are strongly associated with YTHDC2 loss-of-function mutations. The exome sequencing of early-onset POI patients revealed the presence of the missense mutation c.2567C > G (p.P856R) and nonsense mutation c.1129G > T (p.E377) in YTHDC2. The p.P856R mutation is within the helicase-associated domain (HA2 domain), which impairs RNA processing due to the misfolding of RNA helicase conformational flexibility. The nonsense mutation p.E377 truncates the helicase core region [385]. YTHDC2 is essential for the completion of meiosis in mammalian germ cells. Animal studies reveal that the ovaries of female mice lacking Ythdc2 are significantly smaller, with a histology showing the arrest of follicular development at the primordial and primary follicle stages. These follicles cannot continue the secondary or antral stages, exhausting the follicle pool and ovarian reserve. In male germ cells, Ythdc2 absence does not prevent spermatogonial entry into meiosis but leads to arrest at the pachytene-to-diplotene transition, preventing normal diplotene spermatocyte formation and producing abnormal metaphase figures [402]. The use of single-cell transcriptomic analysis reveals that germ cells missing YTHDC2 display mixed mitotic and meiotic transcriptomic profiles after meiotic initiation, indicating the failure to accomplish the mitosis-to-meiosis transition and thus arrests in pre-meiotic [203].

The use of single-cell transcriptomic analysis reveals that germ cells missing YTHDC2 display mixed mitotic and meiotic transcriptomic profiles after meiotic initiation, indicating failure to accomplish the mitosis-to-meiosis transition and thus arrest in pre-meiotic [189]. FTO protects GCs from oxidative stress damage via the miR-642a-5p/FOXO1 signalling axis by stabilising exosomal circular RNA BRCA1 (circBRCA1) [225].

FTO also affects ovarian ageing via m^6^A demethylation. In aged GCs and FTO, downregulation results in abnormal m^6^A-fos mRNA accumulation, increased stability and high expression, causing GC senescence. ROS also activate this pathway through FTO downregulation [403]. In contrast, FTO promotes MMP2 mRNA degradation in a YTHDF2-dependent manner and activates the ERK signalling pathway to delay GC ageing [404]. Essential to primordial follicle pool formation, nuclear-localised FTO is involved in early folliculogenesis. It regulates the alternative splicing of CDK5 via m^6^A demethylation, promoting GC proliferation and cell cycle progression [204]. Additionally, the m^6^A demethylation of long interspersed nuclear element 1 (LINE1) RNA mediated by FTO in embryonic stem cells modifies chromatin’s accessibility, which is involved in regulating oocyte development and early embryogenesis. It awaits to be elucidated at the earliest opportunity that its specific regulatory networks pervade ovarian ageing or the follicular microenvironment [270]. The m^6^A demethylase ALKBH5 is involved in the development of germ cells. The deletion of ALKBH5 significantly enhances m^6^A modification levels in mouse testicular cells and causes the aberrant expression of genes associated with spermatogenesis and apoptosis, impeding male germ cells’ functioning [22]. The mechanisms of ALKBH5 in POI have yet to be defined. Nonetheless, its evolutionarily conserved function to maintain m^6^A modification homeostasis in germ cells suggests that it might regulate ovarian functions.

Iatrogenic POI is a common complication among young cancer survivors. Alkylating agents such as cyclophosphamide (CTX) and selective oestrogen receptor modulators such as tamoxifen (TAM) damage ovarian functioning by disrupting m^6^A homeostasis. Specifically, CTX upregulates GC m^6^A levels in a time- and dose-dependent manner, markedly suppressing demethylase FTO expression while elevating the methyltransferases METTL3 and METTL14 [230]. Maternal tamoxifen exposure during pregnancy downregulates FTO and ALKBH5 while upregulating METTL3 and METTL14, precipitating aberrant primordial follicle assembly and diminishing ovarian reserves in offspring [405]. Environmental endocrine disruptors also interfere with ovarian m^6^A modifications: 4-vinylcyclohexene diepoxide (VCD) reduces global ovarian m^6^A levels in rats [406], whereas the pyrethroid pesticide fenvalerate (FEN) elevates METTL3 and m^6^A reader protein YTHDF2 expression, promoting P-body assembly, disrupting primordial follicle formation and increasing offspring’s POI risk [206].

Beyond m^6^A, m^5^C modifications and their methyltransferases participate in POI pathology. The m^5^C methyltransferase NSUN5 maintains maternal mRNA stability by catalysing m^5^C modifications, ensuring a successful maternal-to-zygotic transition (MZT). Its deletion reduces m^5^C levels, accelerates maternal mRNA degradation and inhibits folliculogenesis, oocyte maturation and embryonic development [121]. TRDMT1 mediates m^5^C modifications in GCs, participating in the DNA damage repair induced by ROS and suppressing oxidative stress-related apoptosis. Functional defects in this enzyme are strongly associated with POI onset and progression [123]. Ac4C modifications also participate in POI pathological processes. In a cyclophosphamide-induced POF mouse model, total mRNA ac4C modification levels in granulosa cells rose markedly, with enhanced ac4C modification at specific sites of senescence inhibitor P16 mRNA. Electroacupuncture treatment reduces ac4C modifications by suppressing NAT10 expression, diminishing P16 mRNA stability and expression, alleviating the inhibition of cyclin-dependent kinase 6 (CDK6) and cyclin D1 (CCND1), restoring granulosa cell proliferation and ameliorating ovarian function. This observation illuminates the reversibility of epitranscriptomic regulation in iatrogenic POI treatment [162].

## 7. RNA Modification Regulation in Gynaecological Benign Diseases

### 7.1. Endometriosis

Endometriosis (EM) refers to the aberrant implantation of endometrial-like tissue outside the uterine cavity as a chronic oestrogen-dependent condition. EM affects approximately 10% of the world’s reproductive-age women. It presents as pelvic pain that progresses and includes infertility. Pathogenesis involves retrograde menstruation, immune evasion, inflammatory microenvironment remodelling and progesterone resistance, culminating in cyclical bleeding, fibrosis and neovascularisation within ectopic lesions [407,408]. Genetic variation alone does not completely determine the disease risk of susceptibility loci identified by genome-wide association studies, implicating the epigenetic regulation of the pathogenesis of the disease. Investigations have confirmed that epigenetic mechanisms modulate the hormonal responsiveness and invasive phenotypes of endometrial cells. Epitranscriptomic regulation is influenced by RNA modifications that illustrate endometriosis’s molecular pathophysiology.

The pathological progression of EMs involves aberrant m^6^A modification profiles. Patients with endometriosis have lower levels of m^6^A in ectopic endometrial tissue due to lower METTL3 expression. This attenuation weakens DiGeorge syndrome critical region gene 8 (DGCR8)-mediated pri-miR-126 maturation, promoting the invasion and migration of endometrial stromal cells (ESCs) [402]. Conversely, METTL3 has a dual regulatory effect on EMs. This enzyme works with YTHDF2 to alter the m^6^A on sirtuin 1 (SIRT1) mRNA, modulating the SIRT1/FoxO3a signalling axis to trigger cellular senescence, inhibiting the migration, invasion and proliferation of ESCs [388]. METTL3 also provides a protective effect by regulating the alternative splicing of lncRNA MIR17HG [409]. Aside from altering the biological behaviour of ESCs, m^6^A participates in immune microenvironment remodelling. Lactic acid derived from ectopic ESCs upregulates METTL3 expression and promotes the m^6^A modification of Tribbles pseudokinase 1 (Trib1), activating the ERK/STAT3 signalling pathway, inducing M2 macrophage polarisation and fostering ectopic lesions’ progression [391]. Other regulatory factors of m^6^A are involved in EM pathology. The expression of METTL14 is significantly decreased in ectopic endometrium from EM patients. The individual or combined loss of METTL14 and METTL3 promotes the proliferation and invasion of ESC via m^6^A-dependent mechanisms [410]. Other regulatory factors of m^6^A are involved in EM pathology. The expression of METTL14 is significantly decreased in an EM patient’s ectopic endometrium. The individual or combined loss of METTL14 and METTL3 promotes the proliferation and invasion of ESC via m^6^A-dependent mechanisms [392].

Patients with EM exhibit the downregulation of the m^6^A demethylase FTO. The overexpression of FTO decreases the m^6^A levels in the mRNA of autophagy-related gene 5 (ATG5), which further increases autophagy, reduces the expression of pyruvate kinase M2 (PKM2) and ultimately reduces the glycolysis, proliferation and migration of ectopic endometrial stromal cells (EESCs) [389]. In terms of regulating the energy metabolism, METTL3 enhances the recognition of m^6^A by the E3 ubiquitin ligase RNF43 and promotes the ubiquitination and degradation of NDUFS1. This inhibits oxidative phosphorylation in ectopic ESCs that modulate energy metabolism and growth in cells [411]. Aberrant m^6^A changes not only promote local EM lesion progression but also greatly contribute to EM-related infertility. Such patients exhibit elevated METTL3 expression and m^6^A levels while the expression of FOXO1 is reduced [11]. Mechanistic investigations demonstrate that METTL3-mediated m^6^A impairs ESC decidualisation capacity through YTHDF2-dependent FOXO1 mRNA degradation pathways, consequently affecting embryo implantation [387]. Hypoxia-induced ALKBH5 upregulation stabilises histone methyltransferase EZH2 mRNA, elevating H3K27me3 levels at decidualisation markers, including IGFBP1 and prolactin (PRL), producing decidualisation defects and embryo implantation failure [390].

Diagnostic applications indicate that the aberrant expression of m^6^A regulatory factors, such as HNRNPA2B1 and HNRNPC, may associate with immune responses in EM and serve as potential biomarkers for diagnoses [412]. M^6^A modification operates through regulating ESC proliferation, invasion, immune microenvironment remodelling, energy metabolism and decidualisation throughout the onset, progression and the infertility associated with EM. Interventions targeting m^6^A-modifying enzymes and downstream effectors may offer therapeutic avenues for EM.

Ac4C modification also participates in the progression of EM. NAT10-mediated ac4C levels are markedly elevated in ectopic endometrial tissue from EM patients. Ac4C advances the proliferation, epithelial–mesenchymal transition (EMT) and cell cycle progression of endometrial epithelial cells by stabilising transforming growth factor-β1 (TGFβ1) mRNA and enhancing target gene expression [413].

### 7.2. Adenomyosis

Adenomyosis refers to the benign invasion of the endometrial glands and stroma into the myometrium [414]. Adenomyosis affects parous perimenopausal women who present abnormal uterine bleeding and severe dysmenorrhea. Imaging techniques widely detect adenomyosis in younger reproductive-age women. This condition is closely associated with miscarriages and infertility. Molecular pathogenesis encompasses dysregulated sex hormone receptor signalling, chronic inflammation, aberrant extracellular matrix deposition and angiogenesis. However, current theories fail to adequately account for the correlations between diverse clinical presentations and imaging phenotypes [415]. Adenomyosis commonly presents with endometriosis and uterine fibroids, which complicates the clinical diagnosis and treatment of pelvic disorders [416]. The current understanding of how adenomyosis develops is limited and utilises classical molecular pathways. A probe into new biological levels, such as epitranscriptomic regulation, is likely to explain adenomyosis by describing the nature of the disease.

The abnormal expression of m^6^A modification and regulators are involved in adenomyosis pathology. Bioinformatics analysis demonstrates that adenomyosis patients specifically display the downregulation of METTL3, ZC3H13, FTO and YTHDC1, resulting in reduced overall m^6^A modification levels in their endometrial tissue [393]. There is a significant negative correlation between the expression levels of IGF1 and D-dopachrome tautomerase (DDT) with METTL3. Such factors may enhance endometrial dysfunctioning by inducing epithelial cell proliferation and migration. Total m^6^A levels are similarly reduced within the myometrium, and 11 m^6^A regulatory factors (including METTL3 and FTO) demonstrate differential expression. Researchers also have identified possible targets, such as cadherin 3 (CDH3), sodium channel β-subunit 4 (SCN4B) and placenta-specific protein 8 (PLAC8), which may be involved in regulating cell adhesion, muscle contraction and the immune response [393].

**Figure 4:**
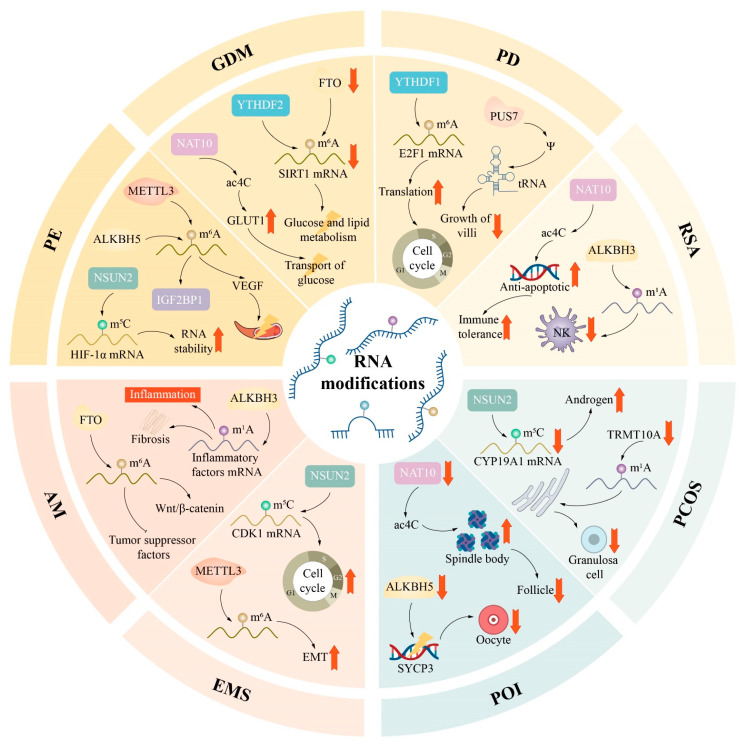
RNA modification regulation in other gynaecological and obstetrical disorders. A. Pregnancy-Related Disorders: including pre-eclampsia (PE), gestational diabetes mellitus (GDM), placental dysfunction (PD), and recurrent spontaneous abortion (RSA). During placental development, m5C and m6A modifications influence trophoblast invasion and angiogenesis via the HIF-1α and VEGFA pathways. In GDM, ac4C modification is involved in glucose metabolism dysregulation by targeting GLUT1. B. Reproductive Endocrine Diseases: including polycystic ovary syndrome (PCOS), primary ovarian insufficiency (POI). In the ovaries, RNA modifications (e.g., m6A, ac4C) regulate steroidogenesis in granulosa cells and meiosis in oocytes. Dysfunctions of NSUN2 or NAT10 are closely linked to hormonal imbalance in PCOS and compromised oocyte quality in POI. C. Benign Gynaecological Diseases: including endometriosis (EMS), adenomyosis (AM). In endometriosis and adenomyosis, m5C and ac4C promote epithelial–mesenchymal transition (EMT) and ectopic invasion by enhancing the expression of CDK1 and Vimentin.

As discussed in previous sections, in reproductive disorders, such as preeclampsia, gestational diabetes, recurrent miscarriage, PCOS and EM, different regulatory patterns of RNA modifications are observed, such as m^6^A, m^5^C, ac4C and m7G. These discussions highlighted the molecular mechanisms of four key modification enzymes, METTL3, FTO, NSUN2 and NAT10, in governing key biological events including trophoblast invasion, maternal–foetal immune tolerance, follicular microenvironment homeostasis and endometrial receptivity that may epitranscriptomically underlie these diseases (Figure 4).

## 8. New Technologies for RNA Modification Detection

Research on RNA modifications has sped up substantially: More than 170 chemical modifications have been identified to date in rRNAs, tRNAs, mRNAs and non-coding RNAs. Changes in the expression of genes require the invention of a high-throughput, accurate detection technique [417,418].

MeRIP-seq (m^6^A RNA immunoprecipitation-sequencing) is an early high-throughput technology that enriches modified RNA fragments using specific antibodies followed by sequencing. Although the method is conceptually simple and broadly applicable, it can only achieve a resolution of approximately 100 bases, making single-base resolution impossible. Antibody-dependent approaches, such as Immunoprecipitation (miCLIP), were developed later at higher resolution. The specificity of the antibody remains problematic, as observed with antibodies used for m1A detection that generated false positives due to their non-specificity with the m7G cap [419,420].

The m^6^A Individual–Nucleotide–Resolution Crosslinking and miCLIP techniques utilise ultraviolet light to create covalent crosslinks between antibodies and modification sites, combining immunoprecipitation and sequencing to achieve single-base resolution. According to relative MeRIP-seq analysis, miCLIP identifies an m^6^A site with low background noise [421,422].

Significant progress has been made in nanopore direct RNA sequencing technology. This platform assesses real-time alterations that occur as RNA moves through protein nanopores and reads unmodified, natural, full-length RNA without reverse transcription or PCR amplification. Hence, it simultaneously identifies several types of modifications while avoiding amplification biases. Early strategies were largely limited to specific modifications; however, recent deep learning frameworks have expanded the areas of application significantly. Omni-RNA modification Characterisation and Annotation (ORCA) uses a domain adversarial learning strategy to capture signals that vary due to mixed chemical stoichiometries and allows the zero-shot detection of diverse modifications such as m^6^A, m^5^C, Ψ and m^1^A. This platform finds types of modifications that were not part of the training data and analyses the synergistic and competitive effects of neighbouring modification sites at the single molecule level [423]. Similarly, DirectRM incorporates base recognition errors and differences in signal features within a multi-label learning architecture, allowing the simultaneous quantification of multiple modifications at the single-molecule level. Next-generation sequencing validation shows that the prediction accuracy of DirectRM for m^6^A sites is substantially higher than classical methods [424]. Using AI-based algorithms can improve accuracy and break away from using pre-trained data, providing more tools to analyse the crosstalk of modification.

Direct RNA sequencing by nanopore technology measures variations in current as RNA molecules are passed through protein nanopores, allowing the reading of natural full-length RNA molecules without the need for reverse transcription or PCR amplification. Current signals will shift due to different modifications and their simultaneous readouts, for example, m^6^A, m^5^C and Ψ, without amplification bias [420]. Nevertheless, this technology still struggles to interpret signals successfully, generating high rates of false positives and optimising algorithms. The accuracy and sensitivity of machine learning methods are continuously in the improvement stage [425].

Enzyme-assisted sequencing uses specific nucleic acid endonucleases or enzyme-catalysed chemical conversions. These methods are limited due to the range of recognition of the enzyme to specific sequence motifs and the limited availability of these enzymes. Chemical labelling sequencing works through reagents such as CMC or bisulfite, inducing reverse transcription to pause for modification site localisation. Chemical treatments that are more aggressive can break nucleic acid chains, while low-abundance modifications require optimisation. Metabolic labelling sequencing characterises newly synthesised RNA through analogue incorporation (e.g., N6-allyl adenosine), which is useful for functional studies but needs matched controls and is not suitable for complex clinical samples [426,427].

Technologies for the detection of RNA modification in single cells reveal cellular heterogeneity. Traditional high-throughput sequencing only shows a populational average, not an individual. Detection methods are becoming more sensitive, gaining greater accuracy. For example, mass spectrometry-based single-cell RNA modification analysis (SNRMA-MS) detects multitudinous modifications in a single neuron to uncover cell-specific modification profiles [428]. Single-cell long-read sequencing also allows the analysis of modifications at single-cell resolution. However, data volume and analysis methods remain challenging [429].

RNA modification detection technologies have unique advantages for different cumulative applications. MeRIP-seq and miCLIP are utilisable for large-scale screening and precise location. Meanwhile, next-generation nanopore sequencing coupled with deep learning (e.g., ORCA and DirectRM) allows multi-modification joint detection and crosstalk analysis, advancing research towards finer cellular resolution using single-cell methodologies. The integration of disparate detection platforms with advanced computational algorithms would increase detection accuracy and widen the applicability that gives insights into the complex regulatory networks of RNA modifications in physiological and pathological processes.

## 9. Conclusions and Future Perspectives

RNA epitranscriptomics has revealed that chemical modifications are widespread and function as critical regulatory hubs in female reproduction. These modifications are intricately involved in the determination of reproductive cell fate, oocyte maturation, embryo implantation and placental development, enabling the precise spatio–temporal control of transcript metabolism throughout reproductive processes. These advances have significantly deepened our understanding of the complexity of female reproduction and have provided new perspectives for elucidating the pathogenesis of gynaecological diseases.

Rapid technological innovation has driven substantial progress in this field. The integration of high-throughput sequencing technologies with single-base resolution detection methods now enables precise mapping and dynamic monitoring of transcriptome-wide RNA modifications. These advances not only facilitate mechanistic studies but also hold promise for clinical translation. Indeed, accumulating clinical evidence suggests that aberrant RNA modification patterns are associated with gynaecological malignancies, recurrent miscarriages and preeclampsia, highlighting their potential as diagnostic biomarkers and therapeutic targets.

Despite these encouraging developments, several important limitations of the current body of research should be acknowledged. Notably, a substantial proportion of the mechanistic insights discussed above are derived from in vitro cell line experiments (e.g., trophoblast cell lines, such as HTR-8/SVneo) and in vivo animal models, particularly murine systems, rather than from direct human clinical studies. While these models provide valuable insights into molecular mechanisms, they may not fully recapitulate the complexity, heterogeneity and physiological context of human reproductive systems.

For instance, differences in gene regulation, the immune microenvironment and endocrine signalling between mouse models and humans may lead to discrepancies in observed phenotypes and therapeutic responses. Similarly, immortalised cell lines often lack the three-dimensional architecture, and cellular interactions present in native tissues, which may limit the extrapolation of findings to clinical settings.

Clinical investigations are often limited by small sample sizes and a lack of multicentre validation, thereby restricting the generalisability of findings. In addition, the current research tends to focus on individual RNA modifications, particularly m6A, with insufficient attention given to the coordinated interplay among multiple modification types, leaving the broader regulatory network incompletely understood.

Another critical challenge lies in the presence of inconsistent and sometimes contradictory findings. For example, the role of ALKBH5 in reproductive disorders remains controversial, with studies suggesting both promotive and context-dependent effects on trophoblast function and pregnancy outcomes. Such discrepancies likely arise from differences in experimental models, disease contexts and downstream molecular targets, underscoring the complexity and context-specific nature of RNA modification-mediated regulation.

Furthermore, the overall strength of the current evidence remains limited. Many conclusions are based on correlative analyses without sufficient causal validation, and high-quality, large-scale clinical studies are still lacking. Therefore, the clinical applicability of RNA epitranscriptomic findings should be interpreted with caution.

Future researchers should prioritise translational approaches, including well-designed prospective clinical studies, patient-derived organoids and human tissue-based validation systems, to bridge the gap between experimental findings and clinical applications. Establishing standardised pipelines for validating RNA modification targets in human cohorts will be essential for advancing these discoveries towards precision medicine.

Meanwhile, an integrated multi-omics approach should be adopted to systematically characterise the interactions among various RNA modifications and to construct a comprehensive regulatory network. Elucidating the crosstalk among writer, eraser and reader proteins will be essential for understanding the coordinated regulation of gene expression in reproductive biology. Systems biology models that integrate transcriptomic, epitranscriptomic and clinical data will further enable the accurate characterisation of disease-associated modification landscapes and support patient stratification.

In addition, the translation of emerging technologies, such as nanopore sequencing, into clinical settings is critical for establishing sensitive and noninvasive platforms for RNA modification detection. Ultimately, precision therapeutic strategies targeting RNA modification enzymes or employing targeted delivery systems may offer novel avenues for restoring cellular homeostasis by modulating epitranscriptomic states.

Despite the remarkable heterogeneity of female reproductive disorders, a careful synthesis of the literature reveals both common principles and context-specific features that govern RNA modification networks across physiological and pathological states.

Common features include: a conserved ‘writer–eraser–reader’ regulatory logic, where the deposition, removal and interpretation of RNA marks operate as an integrated system across virtually all reproductive cell types; functional convergence on key signalling pathways, with m^6^A, m^5^C and ac^4^C modifications frequently converging on PI3K/AKT, Wnt/β-catenin, JAK/STAT and MAPK/ERK cascades to control cell proliferation, dynamics and survival, and differentiation; dynamic reversibility, enabling the rapid adaptation to hormonal fluctuations, metabolic changes and environmental stresses—a feature particularly relevant to cyclic ovarian and endometrial remodelling; broad involvement in non-coding RNA regulation, where RNA modifications orchestrate the stability, processing and function of lncRNAs, circRNAs, miRNAs and piRNAs, thereby amplifying the regulatory reach of the epitranscriptome; and tissue- and cell-type specificity of expression patterns for modification enzymes, explaining how similar molecular events can produce divergent outcomes in different reproductive compartments.

Specific features, conversely, distinguish individual diseases and physiological processes. In gynaecologic cancers, RNA modifications predominantly drive oncogenic pathways by stabilising pro-tumour transcripts (e.g., MYC, FOXM1 and PDGFRB), enhancing the translation of cell cycle regulators (e.g., EIF3C and CDC25B) and reshaping the tumour immune microenvironment through chemokine and immune checkpoint regulation. In pregnancy-related disorders, the focus shifts to trophoblast invasion, angiogenesis and maternal–foetal immune tolerance, with modifications such as m^6^A and m^5^C critically controlling genes such as MYLK, CYR61 and PPARG. In reproductive endocrine disorders, such as PCOS and POI, RNA modifications primarily affect granulosa cell steroidogenesis, insulin signalling and oxidative stress responses, with FTO and METTL3 emerging as central hubs. In benign gynaecological conditions, such as endometriosis and adenomyosis, the epitranscriptome modulates ectopic cell survival, the mesenchymal transition and inflammatory microenvironment remodelling. Finally, during normal physiological processes, RNA modifications ensure precise spatio–temporal control of germ cell development, oocyte meiotic progression, maternal-to-zygotic transition and endometrial receptivity, with each stage characterised by distinct modification landscapes.

Collectively, these observations suggest that while the molecular toolkit of RNA modifications is shared across reproductive contexts, the specific outputs are dictated by cell type, developmental stage and disease context. This duality underscores the importance of moving beyond simplistic ‘oncogene or tumour suppressor’ classifications towards a more nuanced understanding of context-dependent epitranscriptomic regulation.

A critical question in the field is whether the RNA modification patterns described in individual studies align with findings from unbiased genome-wide analyses. The literature reviewed herein demonstrates substantial consistency between targeted mechanistic studies and global epitranscriptomic profiling.

First, MeRIP-seq and m^6^A-seq datasets across multiple gynaecologic cancers consistently reveal that METTL3/METTL14 are frequently dysregulated, with global m^6^A level changes correlating with tumour grade and prognosis. These genome-wide data corroborate functional studies showing that METTL3 drives proliferation, invasion and chemotherapy resistance in cervical, ovarian and endometrial cancers. Similarly, global m^6^A profiling in pregnancy-related disorders has confirmed that altered m^6^A landscapes—particularly at the 5′UTR of trophoblast-associated transcripts—are associated with preeclampsia and foetal growth restriction.

Second, transcriptome-wide mapping of m^5^C and m^1^A has revealed that these modifications are enriched in specific regions (coding sequences for m^5^C, GC-rich regions for m^1^A) and that their regulatory enzymes (NSUN2, ALKBH3, TRMT6) show expression patterns that mirror functional outcomes. For instance, genome-wide m^5^C profiling in cervical cancer has validated NSUN2 as a critical writer, with the hypermethylation of oncogenic transcripts (KRT13, LRRC8A) identified as a recurrent feature across patient cohorts.

Third, integrated multi-omics analyses (combining RNA-seq, MeRIP-seq and clinical data) from large-scale resources, such as The Cancer Genome Atlas (TCGA) and Gene Expression Omnibus (GEO), have consistently identified RNA modification regulators as prognostic biomarkers. Prognostic models based on m^6^A-, m^7^G- or m^5^C-related gene signatures have been validated across independent cohorts, reinforcing the clinical relevance of these modifications. Moreover, pathway enrichment analyses from these genome-wide studies converge on the same signalling axes (PI3K/AKT, Wnt, JAK/STAT) identified in mechanistic studies, providing orthogonal validation.

Fourth, genome-wide association studies (GWAS) have identified genetic variants in RNA modification genes—such as FTO rs9939609 in PCOS and YTHDC2 loss-of-function mutations in POI—that are significantly associated with disease susceptibility, establishing a direct link between the epitranscriptomic machinery and disease aetiology at the population level.

Notably, discrepancies exist. Single-gene functional studies occasionally report contradictory findings regarding the directionality of enzyme activity (e.g., ALKBH5 acting as either a tumour suppressor or oncogene), which may reflect cell-type specificity, experimental conditions or technical differences that are not always resolved in global profiling datasets. Future studies integrating single-cell epitranscriptomic sequencing with functional perturbation will be essential to reconcile these discrepancies.

In summary, the preponderance of evidence indicates that the RNA modification patterns observed in targeted mechanistic studies are broadly consistent with unbiased genome-wide analyses. This convergence strengthens the credibility of current findings and supports the continued translation of epitranscriptomic discoveries towards diagnostic and therapeutic applications.

## Figures and Tables

**Table 1:** Overview of key regulatory proteins involved in major RNA modifications.

RNA Modification	Class	Regulator	Catalytic Activity	Subcellular Localisation	Functions	Ref
m^6^A	Writer	METTL3	Yes (m^6^A methyltransferase; catalytic subunit)	Nucleus	Core catalytic component of the m^6^A writer complex. Forms a heterodimer with METTL14 to deposit m^6^A at RRACH motifs, thereby regulating pre-mRNA splicing, mRNA stability and translation.	[17]
		METTL14	No (degenerate catalytic site; primarily mediates RNA binding and substrate recognition)	Nucleus	Facilitates substrate recognition and stabilises the METTL3-containing writer complex, contributing to the specificity of m^6^A deposition.	[18]
		METTL16	Yes (m^6^A methyltransferase)	Nucleus	Installs m^6^A on U6 snRNA and selected mRNAs (for example, MAT2A), linking m^6^A to splicing regulation and feedback control of methionine/SAM metabolism.	[19]
		WTAP	No (regulatory/scaffold protein)	Nucleus (nuclear speckles)	Recruits and localises METTL3/METTL14 to nuclear speckles, regulating complex assembly and substrate selection.	[20]
	Eraser	FTO	Yes (Fe(II)/α-KG-dependent demethylase)	Nucleus/Cytoplasm	Fe(II)/α-ketoglutarate-dependent demethylase that removes m^6^A and related marks (for example, m^6^Am), thereby influencing mRNA stability and translation.	[21]
		ALKBH5	Yes (Fe(II)/α-KG-dependent demethylase)	Nucleus	Removes m^6^A from RNA, modulating nuclear export and transcript stability; implicated in reproduction and diverse disease processes.	[22]
	Reader	YTHDF1	No (m^6^A-binding protein)	Cytoplasm	Binds m^6^A-modified transcripts and promotes translation efficiency and protein output.	[23]
		YTHDF2	No (m^6^A-binding protein)	Cytoplasm (P-bodies)	Recognises m^6^A marks and promotes target mRNA deadenylation and decay, including via recruitment of the CCR4-NOT complex.	[24]
		YTHDF3	No (m^6^A-binding protein)	Cytoplasm	Coordinates with YTHDF1 and YTHDF2 to fine-tune translation and mRNA decay.	[25]
		YTHDC1	No (m^6^A-binding protein)	Nucleus	Nuclear m^6^A reader that regulates alternative splicing and nuclear export of m^6^A-marked transcripts.	[26]
		YTHDC2	No (reader; contains an ATP-dependent helicase domain)	Cytoplasm	m^6^A-binding RNA helicase implicated in translational control and mRNA turnover; critical for gametogenesis.	[27]
		IGF2BP1	No (m^6^A-binding protein)	Cytoplasm	‘Stabilising’ m^6^A reader that enhances the stability and translation of m^6^A-marked target transcripts.	[28]
		IGF2BP2	No (m^6^A-binding protein)	Cytoplasm	Binds m^6^A-modified RNAs to promote mRNA stability and translation.	[29]
		IGF2BP3	No (m^6^A-binding protein)	Cytoplasm	Binds m^6^A-modified RNAs to promote mRNA stability and translation.	[30]
		HNRNPC	No (RNA-binding protein)	Nucleus	Binds RNA structural switches adjacent to m^6^A (‘m^6^A-switch’ mechanism), thereby modulating splicing and other post-transcriptional processes.	[31]
A-to-I editing	Editor	ADAR1	Yes (adenosine deaminase; A → I editing)	Nucleus/Cytoplasm	Catalyses adenosine-to-inosine editing in double-stranded RNA, reducing endogenous dsRNA immunogenicity and influencing recoding, splicing and RNA stability.	[32]
		ADAR2 (ADARB1)	Yes (adenosine deaminase; A → I editing)	Nucleus	Predominantly nuclear editor that mediates transcriptome recoding and contributes to tissue homeostasis.	[33]
		ADAR3 (ADARB2)	No (lacks deaminase activity; regulatory)	Nucleus/Cytoplasm	Generally catalytically inactive; binds RNA and can modulate or inhibit editing mediated by other ADAR enzymes.	[34]
Ψ (pseudouridylation)	Writer	PUS7	Yes (pseudouridine synthase)	Nucleus/Cytoplasm	Standalone pseudouridine synthase that catalyses U → Ψ in multiple RNA species (including mRNA and tRNA), contributing to stress-responsive translation and post-transcriptional regulation.	[35]
		PUS1	Yes (pseudouridine synthase)	Nucleus/Mitochondria	Catalyses pseudouridylation at multiple sites across RNA classes, including mitochondrial mRNA and tRNA, affecting RNA stability and translation.	[36]
		DKC1 (H/ACA snoRNP)	Yes (catalytic subunit of H/ACA snoRNP pseudouridine synthase)	Nucleolus/Nucleus	Core component of guide RNA-dependent pseudouridylation machinery. Primarily modifies rRNA and snRNA and supports ribosome biogenesis and telomerase function.	[37]
	Reader	PFN1	No (Ψ-binding protein)	Cytoplasm	Pseudouridine-RNA binding protein (reader) that selectively binds Ψ-containing RNAs and modulates downstream post-transcriptional processes.	[38]
		PABPC1	No (RNA-binding protein)	Cytoplasm	Reported to preferentially bind Ψ-containing RNAs and may affect poly(A)+ mRNA stability and translation (putative reader).	[39]
m^5^C	Writer	NSUN2	Yes (m^5^C methyltransferase)	Nucleus/Cytoplasm	Major mRNA/tRNA m^5^C writer that regulates RNA stability, translation and stress responses.	[40]
		NSUN6	Yes (m^5^C methyltransferase)	Cytoplasm	Installs m^5^C at specific sites in mRNA and tRNA, influencing translation efficiency.	[41]
		DNMT2/TRDMT1	Yes (RNA methyltransferase)	Cytoplasm	Primarily methylates tRNA C38 to form m^5^C, maintaining tRNA stability and facilitating adaptation to cellular stress.	[42]
	Eraser	TET1/2/3	Yes (dioxygenases; oxidise m^5^C to hm^5^C and related derivatives)	Nucleus	Oxidises m^5^C to hm^5^C and related derivatives, providing a route for reversible epitranscriptomic regulation.	[43]
		ALKBH1	Yes (Fe(II)/α-KG-dependent dioxygenase/demethylase)	Nucleus/Mitochondria	Fe(II)/α-ketoglutarate-dependent enzyme implicated in dynamic regulation of RNA m^5^C (and related) modifications.	[44]
	Reader	ALYREF	No (m^5^C-binding protein)	Nucleus	Nuclear m^5^C reader that promotes export of m^5^C-modified mRNAs.	[45]
		YBX1	No (m^5^C-binding protein)	Cytoplasm	Cytoplasmic m^5^C reader that enhances the stability of m^5^C-marked mRNAs and promotes translation.	[46]
m^7^G	Writer	METTL1/WDR4	Yes (METTL1 catalytic; WDR4 cofactor)	Nucleus/Cytoplasm	Installs internal m^7^G on tRNA (G46) and subsets of mRNA/pri-miRNA, promoting RNA stability, nuclear export and translation.	[47]
		WBSCR22/TRMT112	Yes (WBSCR22 catalytic; TRMT112 cofactor)	Nucleolus	Installs m^7^G at 18S rRNA G1639, contributing to ribosome biogenesis and maturation.	[47]
		RNMT/RAM	Yes (RNMT catalytic; RAM activating cofactor)	Nucleus	Catalyses formation of the 5′ mRNA cap m^7^G, influencing transcript maturation, nuclear export and translation initiation.	[48]
	Reader	eIF4E	No (m^7^G cap-binding protein)	Cytoplasm/Nucleus	Recognises the m^7^G cap and initiates cap-dependent translation; also participates in cap-associated nuclear export pathways.	[49]
		NCBP1/NCBP2 (CBC)	No (m^7^G cap-binding complex)	Nucleus	Nuclear cap-binding complex that binds the m^7^G cap to promote pre-mRNA splicing and nuclear export.	[50]
m^1^A	Writer	TRMT6/TRMT61A	Yes (TRMT61A catalytic; TRMT6 structural)	Nucleus/Cytoplasm	Catalyses m^1^A58 formation in tRNAs, influencing RNA structure, stability and translation.	[51]
		TRMT10C (MRPP1)	Yes (mitochondrial tRNA methyltransferase)	Mitochondria	Installs m^1^A in mitochondrial tRNAs (as part of mitochondrial RNase P), supporting mitochondrial gene expression and translation.	[52]
		TRMT61B	Yes (mitochondrial tRNA methyltransferase)	Mitochondria	Installs m^1^A in mitochondrial tRNAs and helps maintain mitochondrial translational homeostasis.	[53]
		NML	Yes (rRNA methyltransferase)	Nucleolus	Catalyses m^1^A formation in rRNA and links ribosome biogenesis to metabolic adaptation.	[53]
	Eraser	ALKBH1	Yes (Fe(II)/α-KG-dependent dioxygenase/demethylase)	Nucleus/Mitochondria	Removes m^1^A (and related marks) from tRNA and mitochondrial RNAs, modulating translation and stress responses.	[53]
		ALKBH3	Yes (Fe(II)/α-KG-dependent demethylase)	Nucleus/Cytoplasm	Demethylates m^1^A (and m^3^C) in RNA, influencing RNA stability and translation and contributing to oncogenic processes in some contexts.	[54]
		ALKBH7	Yes (mitochondrial demethylase; reported to remove m^1^A and related marks)	Mitochondria	Mitochondria-associated demethylase implicated in RNA processing and metabolic stress responses.	[55]
		FTO	Yes (Fe(II)/α-KG-dependent demethylase)	Nucleus/Cytoplasm	Can demethylate m^1^A in selected substrates (in addition to m^6^A/m^6^Am), affecting RNA fate.	[56]
	Reader	YTHDF1/2/3	No (reader proteins)	Cytoplasm	Reported to recognise m^1^A in a substrate-dependent manner and regulate translation and/or decay of marked RNAs.	[57]
		YTHDC1	No (reader protein)	Nucleus	Nuclear YTH-domain reader that can recognise m^1^A and/or m^6^A in a context-dependent manner, regulating splicing and nuclear export.	[58]
ac^4^C	Writer	NAT10	Yes (acetyltransferase)	Nucleolus/Nucleus/Cytoplasm	RNA acetyltransferase that installs ac^4^C on tRNAs, 18S rRNA and subsets of mRNAs, enhancing translational fidelity and modulating mRNA stability.	[59]
mcm^5^s^2^U	Writer	TRM9L and ALKBH8	Partial (ALKBH8 is catalytic)	Cytoplasm (tRNA U34)	ALKBH8 catalyses cm^5^U → mcm^5^U and subsequent hydroxylation at the tRNA U34 wobble position. mcm^5^U is a key intermediate for generating mcm^5^s^2^U and related U34 modifications, thereby influencing decoding and translation.	[60]

Abbreviations: ac^4^C, N4-acetylcytidine; ADAR, adenosine deaminase acting on RNA; A-to-I, adenosine-to-inosine; α-KG, alpha-ketoglutarate; CBC, cap-binding complex; cm^5^U, 5-carboxymethyluridine; DKC1, dyskerin pseudouridine synthase 1; dsRNA, double-stranded RNA; Fe(II), divalent iron ion; H/ACA snoRNP, H/ACA-type small nucleolar ribonucleoprotein; hm^5^C, 5-hydroxymethylcytidine; m^1^A, N1-methyladenosine; m^5^C, 5-methylcytidine; m^6^A, N6-methyladenosine; m^6^Am, N6,2′-O-dimethyladenosine; m^7^G, N7-methylguanosine; mcm^5^s^2^U, 5-methoxycarbonylmethyl-2-thiouridine; mcm^5^U, 5-methoxycarbonylmethyluridine; mRNA, messenger RNA; mtRNase P, mitochondrial RNase P; P-bodies, processing bodies; pri-miRNA, primary microRNA; Ψ, pseudouridine; RRACH, consensus sequence motif for m^6^A deposition (R = A/G; A = m^6^A; H = A/C/U); rRNA, ribosomal RNA; SAM, S-adenosylmethionine; snRNA, small nuclear RNA; tRNA, transfer RNA; U34, uridine at the wobble position 34 of tRNA.

**Table 2:** Regulatory roles of RNA modifications in gynecologic cancers, pregnancy-associated disorders, reproductive endocrine disorders and benign gynecologic diseases.

Disease Category	Condition	Specimen/Target	Methods	RNA Modification (Enzyme/Protein)	Pathway/Target	Biological Effect	Ref.
Gynecologic cancers	Cervical cancer	Cervical cancer cell lines (SiHa, HeLa, C33A)	MeRIP-seq; qRT-PCR; Western blotting; cell proliferation and invasion assays	m^6^A/METTL3 (writer)	METTL3-mediated m^6^A stabilises lncRNA FOXD2-AS1, which recruits LSD1 to the p21 promoter and represses p21.	Promotes cervical cancer cell proliferation and migration.	[84]
		Cervical cancer tissues; SiHa and HeLa cell lines	MeRIP-seq; miRNA sequencing; luciferase reporter assay	m^6^A/METTL3 (writer)	METTL3-dependent m^6^A suppresses the tumour-suppressive miR-193b, leading to CCND1 overexpression.	Promotes cervical cancer invasion and deep stromal infiltration.	[91]
		Cervical cancer cell lines (SiHa, CaSki)	RNA-seq; MeRIP-seq; ferroptosis assays	m^6^A/METTL3-KRAS axis (regulated by miR-30c-5p)	miR-30c-5p inhibits METTL3, reducing m^6^A on KRAS mRNA and inducing ferroptosis.	Suppresses cervical cancer growth and metastasis by triggering ferroptosis.	[92]
		Cervical cancer cell lines (SiHa, HeLa); nude mouse xenograft model	RIP-seq; circRNA sequencing; luciferase reporter assays	m^6^A/IGF2BP2 (reader)—circARHGAP12	m^6^A-modified circARHGAP12 is recognised by IGF2BP2, enhancing FOXM1 mRNA stability.	Promotes cervical cancer cell proliferation and migration.	[87]
		Cervical cancer tissues; SiHa and HeLa cell lines	MeRIP-qPCR; RNA immunoprecipitation (RIP); invasion and migration assays	m^6^A/ALKBH5 (eraser)—circCCDC134	ALKBH5 demethylation decreases circCCDC134 stability; circCCDC134 sponges miR-503-5p, thereby promoting HIF1A transcriptional activation.	Promotes cervical cancer invasion and metastasis.	[89]
		Cervical cancer tissues and cell lines; nude mouse model	MeRIP-seq; piRNA sequencing; Western blotting	m^6^A/METTL14 (writer)—piRNA-14633	piRNA-14633 enhances METTL14 mRNA stability, resulting in upregulation of CYP1B1.	Promotes cervical cancer cell proliferation, migration and invasion.	[94]
		Cervical cancer tissues; HeLa and SiHa cell lines	m^5^C-RIP-seq; RNA bisulfite sequencing; Western blotting	m^5^C/NSUN2 (writer); YBX1 (reader)	NSUN2 catalyses m^5^C on LRRC8A mRNA, which is recognised by YBX1 to stabilise the transcript.	Suppresses apoptosis and promotes cervical cancer cell proliferation and metastasis.	[125]
		Cervical cancer tissues; SiHa and HeLa cell lines; tumour immune microenvironment analyses	acRIP-seq; RNA-seq; metabolomics; immunohistochemistry	ac^4^C/NAT10 (writer)	NAT10 installs ac^4^C on FOXP1 mRNA, driving glycolytic rewiring (Warburg effect) and immunosuppression.	Promotes malignant progression and shapes an immunosuppressive microenvironment in cervical cancer.	[160]
	Ovarian cancer	Ovarian cancer cell lines (SKOV3, A2780); nude mouse xenograft model	MeRIP-seq; miRNA sequencing; luciferase reporter assays	m^6^A/METTL3 (writer)	METTL3-mediated m^6^A promotes maturation of pri-miR-126-5p, suppressing PTEN and activating PI3K-Akt-mTOR signalling.	Promotes ovarian cancer proliferation and progression.	[68]
		Ovarian cancer tissues; SKOV3 and A2780 cell lines; immune cell co-culture	Flow cytometry; MeRIP-seq; ELISA; immunohistochemistry	m^6^A/METTL3 (writer)—myeloid-derived suppressor cells (MDSCs)	Tumour cell METTL3-dependent m^6^A promotes recruitment of MDSCs, suppressing CD8+ T-cell responses.	Facilitates immune evasion and tumour progression in ovarian cancer.	[69]
		Ovarian cancer cell lines (SKOV3, HO8910); subcutaneous xenograft model	MeRIP-seq; Western blotting; tumoursphere formation assays	m^6^A/METTL3 (writer)—lncRNA LINC00857	METTL3-mediated m^6^A stabilises LINC00857 and activates YAP-TEAD signalling.	Enhances cancer stem-like properties and metastatic capacity of ovarian cancer cells.	[85]
		Cisplatin-resistant ovarian cancer cell lines (A2780/DDP, SKOV3/DDP)	MeRIP-seq; circRNA-seq; cisplatin resistance assays	m^6^A/IGF2BP1 (reader)—circPLPP4	METTL3-dependent m^6^A enables IGF2BP1 to stabilise circPLPP4, which sponges miR-136, increasing PIK3R1 and activating PI3K-AKT signalling.	Induces cisplatin resistance in ovarian cancer cells.	[381]
		Ovarian cancer tissues and cell lines; hypoxia model	MeRIP-qPCR; Western blotting; Warburg effect assays	m^6^A/WTAP (writer)—HIF-1α/miR-200/HK2 axis	Hypoxia-induced HIF-1α upregulates WTAP, which interacts with DGCR8 to promote pri-miR-200 maturation, increasing HK2 and enhancing the Warburg effect.	Promotes ovarian cancer proliferation, invasion and metastasis and is associated with poor prognosis.	[382]
		Ovarian cancer cell lines (SKOV3, HO8910); intraperitoneal tumour model	RNA immunoprecipitation (RIP); Western blotting; mitochondrial function assays; apoptosis analyses	m^6^A/YTHDC1 (reader)—piR-26441	piR-26441 binds YTHDC1 and prevents TRIM56-mediated degradation of YTHDC1, enhancing m^6^A-dependent decay of TSFM mRNA, reducing oxidative phosphorylation and increasing ROS to trigger apoptosis.	Inhibits ovarian cancer cell proliferation and tumour formation in vivo.	[96]
		Plasma and tumour tissues from ovarian cancer patients; ovarian cancer cell lines	Mass spectrometry for pseudouridine (Ψ); immunohistochemistry; cell proliferation assays	Ψ (pseudouridylation)/PUS7	PUS7-mediated mRNA pseudouridylation influences RNA stability and translation.	High PUS7 expression promotes ovarian cancer cell proliferation; elevated plasma Ψ may serve as a potential early diagnostic biomarker.	[115]
		Ovarian and breast cancer cell lines; macrophage co-culture	PAR-CLIP; m^1^A-RIP; Western blotting; invasion assays	m^1^A/ALKBH3 (eraser)	ALKBH3 removes m^1^A from GC-rich regions of CSF1 mRNA, extending CSF1 mRNA half-life and increasing macrophage recruitment.	Promotes macrophage recruitment and cancer cell invasion in the ovarian tumour microenvironment.	[144]
		Ovarian cancer tissues; SKOV3 and HO8910 cell lines	acRIP-seq; RNA-seq; fatty-acid metabolism assays	ac^4^C/NAT10 (writer)	m^6^A-driven NAT10 translation enhances ac^4^C modification of ACOT7 mRNA, rewiring fatty-acid metabolism to suppress ferroptosis.	Promotes ovarian tumourigenesis and tumour progression.	[88]
	Endometrial cancer	Endometrial cancer cell lines (Ishikawa, HEC-1A); tumour tissues	MeRIP-seq; RNA immunoprecipitation (RIP); Western blotting; JAK/STAT pathway assays	m^6^A/IGF2BP2 (reader)—circCHD7	m^6^A-modified circCHD7 binds IGF2BP2, enhancing PDGFRB mRNA stability and activating JAK/STAT signalling.	Promotes endometrial cancer cell proliferation and progression.	[144]
		TCGA endometrial cancer cohort; immune infiltration datasets	Bioinformatics; m^7^G scoring; drug sensitivity prediction	m^7^G/METTL1; WDR4	m^7^G-related lncRNA/miRNA networks are associated with immune infiltration phenotypes and predicted drug sensitivity.	An m^7^G-related molecular signature may enable prognostic stratification in endometrial cancer.	[134]
		TCGA endometrial cancer cohort	Bioinformatics; Cox regression; immune infiltration analyses	m^1^A/TRMT6, TRMT61A, ALKBH3, YTHDC1, YTHDF2	m^1^A-related lncRNAs were used to construct a prognostic model linked to immune infiltration phenotypes and drug sensitivity.	An m^1^A-related lncRNA signature predicts prognosis and immune response in endometrial cancer.	[147]
	Choriocarcinoma	Choriocarcinoma cell lines (JAR, JEG-3); nude mouse xenograft model	MeRIP-seq; Northern blotting; angiogenesis assays; ELISA	m^6^A/METTL3 (writer)—miR-935/Cx43	METTL3 promotes m^6^A-dependent maturation of pri-miR-935, increasing miR-935 to repress connexin 43 (Cx43/GJA1).	Promotes choriocarcinoma cell proliferation, migration, invasion and angiogenesis.	[93]
Pregnancy-associated disorders	Preeclampsia	Early-onset preeclampsia placental tissues; trophoblast cell line HTR8/SVneo	MeRIP-qPCR; Western blotting; invasion assays; immunohistochemistry	m^6^A/WTAP (writer)—IGF2BP1/HMGN3 axis	WTAP dysregulation alters m^6^A modification of HMGN3 mRNA, which is recognised by IGF2BP1, impairing trophoblast invasion.	Impaired trophoblast invasion contributes to the pathogenesis of early-onset preeclampsia.	[345]
		Preeclampsia placental tissues; trophoblast cell lines HTR8/SVneo and BeWo	MeRIP-seq; ALKBH5 knockdown; Western blotting	m^6^A/ALKBH5 (eraser)—CYR61	ALKBH5 removes m^6^A from CYR61 mRNA, decreasing CYR61 expression and suppressing trophoblast invasion.	ALKBH5-mediated regulation of CYR61 m^6^A is implicated in preeclampsia.	[268]
		Preeclampsia placental tissues; trophoblast cell lines	Transcriptome sequencing; MeRIP-seq; Western blotting	m^6^A/METTL3 (writer)	METTL3 regulates m^6^A modification of trophoblast-associated mRNAs, affecting trophoblast proliferation, migration and invasion.	METTL3 dysregulation contributes to placental dysfunction in preeclampsia.	[344]
	Gestational diabetes mellitus (GDM)	Placental tissues from women with GDM and normoglycaemic controls	MeRIP-seq; RNA-seq; MazF-qPCR; Western blotting	m^6^A/METTL3 (writer)—BAMBI/INSR/IRS1	Global m^6^A levels are reduced in GDM placenta. Decreased m^6^A on BAMBI is associated with reduced BAMBI expression, and decreased m^6^A on INSR/IRS1 is linked to impaired insulin signalling.	Placental m^6^A downregulation and reduced m^6^A on insulin receptor pathway genes are associated with GDM pathogenesis.	[361]
		Placental tissues from women with type 2 diabetes mellitus; trophoblast cell lines BeWo and JEG3	MeRIP-seq; RNA-seq; immunohistochemistry; qRT-PCR; Western blotting	m^6^A/FTO (eraser) and METTL3 (writer)—SIK1/GLUTs	High glucose increases FTO, reducing m^6^A on SIK1 and lowering SIK1 mRNA stability to impair syncytialisation. METTL3 upregulation via mTOR increases GLUT expression, leading to dysregulated placental glucose transport.	Hyperglycaemia-induced FTO and METTL3 dysregulation impairs trophoblast syncytialisation and perturbs placental glucose transport.	[365]
	Placental dysfunction	Trophoblast cell lines HTR8/SVneo and BeWo; Wtap knockout mice	MeRIP-seq; invasion and migration assays; placental developmental phenotyping	m^6^A/WTAP (writer)—IGF2BP1/HMGN3 axis	WTAP-dependent m^6^A promotes activation of the IGF2BP1-HMGN3 axis to enhance trophoblast migration and invasion.	Placenta-specific Wtap knockout causes placental developmental defects in mice.	[346]
		Trophoblast cell lines with METTL14 overexpression or knockdown	Western blotting; apoptosis assays; autophagy flux analyses	m^6^A/METTL14 (writer)—FOXO3a	METTL14 overexpression elevates m^6^A and increases trophoblast apoptosis and autophagy; FOXO3a inhibition partially rescues these defects.	The METTL14-m^6^A axis regulates trophoblast survival and may influence placental function.	[349]
	Miscarriage	Trophoblasts from patients with recurrent spontaneous abortion (RSA)	MeRIP-seq; Western blotting; TGF-β pathway assays	m^6^A/FTO (eraser)—MEG3/TGF-β	FTO removes m^6^A from the lncRNA MEG3, destabilising MEG3 transcripts and activating TGF-β signalling.	FTO-mediated regulation of MEG3 stability and TGF-β signalling is associated with trophoblast dysfunction in RSA.	[160]
		Decidual tissues from patients with recurrent implantation failure (RIF); endometrial stromal cells	MeRIP-seq; decidualisation assays; Western blotting	m^6^A/METTL3 (writer)—HOXA10	METTL3 overexpression increases m^6^A on HOXA10 mRNA, reducing HOXA10 expression and impairing embryo implantation.	METTL3-mediated m^6^A repression of HOXA10 contributes to recurrent implantation failure.	[251]
Reproductive endocrine disorders	Polycystic ovary syndrome (PCOS)	Peripheral blood; population-based genomics studies	Genome-wide association studies (GWAS); meta-analysis; genotyping	m^6^A/FTO polymorphism (rs9939609)	FTO genetic variation is linked to altered m^6^A demethylation activity and dysregulated lipid and androgen metabolism.	FTO variants are significantly associated with PCOS susceptibility and androgen levels, independent of obesity.	[383]
		Granulosa cells from women with PCOS; KGN and COV434 cell lines	MeRIP-qPCR; Western blotting; immunohistochemistry; androgen measurements	m^6^A/FTO (eraser)—FLOT2/androgen axis	FTO demethylates and stabilises FLOT2 mRNA, increasing FLOT2 and promoting insulin resistance in granulosa cells. Dihydrotestosterone (DHT) upregulates FTO in a positive-feedback loop, exacerbating hyperandrogenism.	The FTO-FLOT2 axis contributes to granulosa-cell dysfunction in PCOS and may aggravate hyperandrogenism.	[227]
		Granulosa cells from women with PCOS; KGN cell line	MeRIP-seq; Western blotting; mitochondrial function assays; glycolysis measurements	m^6^A/YTHDF2 (reader)—MSS51/mitochondria	Upregulated YTHDF2 binds MSS51 mRNA and promotes m^6^A-dependent decay, leading to mitochondrial dysfunction and aberrant glycolysis.	YTHDF2 overexpression drives mitochondrial and metabolic dysfunction in PCOS granulosa cells.	[384]
		Luteinised granulosa cells from non-obese women with PCOS	MeRIP-qPCR; Western blotting; FOXO3 pathway analyses	m^6^A/YTHDF2 (reader)—FOXO3	YTHDF2 is increased and promotes m^6^A-dependent degradation of FOXO3 mRNA, reducing FOXO3 and disrupting cellular homeostasis.	The YTHDF2-FOXO3 axis contributes to granulosa-cell dysfunction in non-obese PCOS.	[228]
		Granulosa cells from women with PCOS	Transcriptome sequencing; A-to-I editing site analyses	A-to-I editing/ADARB1 (ADAR2)	Reduced ADARB1 expression decreases A-to-I editing and alters expression of genes involved in granulosa-cell dysfunction.	Dysregulated A-to-I RNA editing is implicated in ovarian dysfunction in PCOS.	[106]
		Peripheral blood and granulosa cells from women with PCOS	A-to-I editing site analyses; RNA-seq; Western blotting; ROC analyses	A-to-I editing/ADAR1—EIF2AK2/MAPK axis	ADAR1-mediated A-to-I editing at a specific EIF2AK2 site (chr2:37100559) increases EIF2AK2 expression and activates MAPK signalling.	Exacerbates PCOS pathology; this editing site shows diagnostic potential (AUC = 0.9167).	[108]
	Primary ovarian insufficiency (POI)	Peripheral blood DNA from patients with POI; mouse POI model	Next-generation sequencing; in silico structural modelling; immunohistochemistry	m^6^A/YTHDC2 (reader)—meiosis/ovarian reserve	Pathogenic YTHDC2 variants (p.P856R and p.E377) impair RNA helicase activity, disrupting oocyte meiotic programmes and causing follicle loss.	YTHDC2 dysfunction is directly associated with early-onset POI and represents a genetic cause of human POI.	[385]
		Granulosa cells from women with POI; cyclophosphamide (CTX)-induced POI mouse model	MeRIP-seq; immunohistochemistry; global m^6^A quantification; Western blotting	m^6^A/FTO (eraser)	Reduced FTO expression increases m^6^A levels in granulosa cells, decreasing proliferation and increasing apoptosis; CTX further elevates m^6^A.	FTO protects granulosa-cell function; low FTO is associated with reduced ovarian reserve and increased POI risk.	[189]
		Conditional Nat10 knockout mice (Stra8-Cre; Zp3-Cre); granulosa cells from women with POI	acRIP-seq; chromosome spread assays; RNA stability analyses	ac^4^C/NAT10 (writer)—meiosis/maternal mRNAs	NAT10 loss abolishes ac^4^C, impairs CCR4-NOT deadenylase activity and disrupts maternal mRNA decay, leading to meiotic arrest at the pachytene stage.	NAT10 deficiency arrests follicles at the primary follicle stage and contributes to premature ovarian insufficiency.	[157]
		Granulosa cells in premature ovarian failure; TRDMT1 knockdown cell model	m^5^C-RIP; Western blotting; γH2AX detection; comet assays	m^5^C/TRDMT1 (DNMT2)—DNA damage repair	TRDMT1 dysregulation reduces tRNA m^5^C, compromising DNA damage repair capacity and increasing granulosa-cell apoptosis.	TRDMT1-mediated m^5^C helps maintain genomic integrity in granulosa cells; its loss is implicated in POI.	[123]
Benign gynecologic diseases	Endometriosis	Eutopic endometrial stromal cells (ESCs) from patients with endometriosis	MeRIP-seq; in vitro decidualisation; Western blotting; immunohistochemistry	m^6^A/METTL3 (writer); YTHDF2 (reader)—FOXO1	METTL3 upregulation increases m^6^A on FOXO1 mRNA, which is recognised by YTHDF2 for degradation, reducing FOXO1 and impairing decidualisation.	The METTL3-YTHDF2-FOXO1 axis impairs decidualisation and contributes to endometriosis-associated infertility.	[386]
		Eutopic endometrial stromal cells (ESCs) from patients with endometriosis; decidualisation model	Western blotting; immunohistochemistry for E-cadherin and vimentin	m^6^A/METTL3 (writer)—MET/endometrial receptivity	METTL3 upregulation suppresses E-cadherin and upregulates vimentin/Slug, inhibiting mesenchymal-to-epithelial transition (MET).	METTL3-mediated inhibition of MET reduces endometrial receptivity and may contribute to endometriosis-related infertility.	[387]
		Ectopic endometrial stromal cells (ESCs) from patients with endometriosis; cellular senescence model	MeRIP-seq; Western blotting; senescence marker assays; invasion assays	m^6^A/METTL3 (writer); YTHDF2 (reader)—SIRT1/senescence	Reduced METTL3 decreases m^6^A on SIRT1 mRNA, diminishing YTHDF2-mediated decay and stabilising SIRT1, thereby increasing cellular senescence and limiting ectopic implantation.	The METTL3-YTHDF2 axis modulates cellular senescence in ectopic ESCs and is linked to lesion progression.	[388]
		Ectopic endometrial stromal cells (ESCs); mouse peritoneal endometriosis model	MeRIP-qPCR; Western blotting; glycolysis assays; autophagy flux analyses	m^6^A/FTO (eraser)—PKM2/ATG5/glycolysis	Reduced FTO increases m^6^A on PKM2 mRNA, elevating PKM2; PKM2 negatively correlates with ATG5, promoting glycolysis and suppressing autophagy.	FTO suppresses glycolysis and promotes autophagy to counteract lesion dissemination in endometriosis.	[389]
		Eutopic endometrial stromal cells (ESCs) from patients with endometriosis; hypoxia model	MeRIP-seq; ChIP-seq (H3K27me3); Western blotting; decidualisation assays	m^6^A/ALKBH5 (eraser)—EZH2/H3K27me3/decidualisation	Hypoxia induces ALKBH5, which stabilises EZH2 mRNA and increases H3K27me3, silencing decidual markers (for example, IGFBP1 and PRL).	ALKBH5 impairs decidualisation via m^6^A-epigenetic crosstalk and may contribute to endometriosis-associated infertility.	[390]
		Ectopic endometrial stromal cells (ESCs); macrophage co-culture	ELISA; Western blotting; flow cytometry for M2 polarisation markers	m^6^A/METTL3 (writer)—lactate/Trib1/ERK/STAT3	Glycolysis-derived lactate activates METTL3; METTL3-mediated m^6^A on Trib1 activates ERK/STAT3 signalling and drives M2 macrophage polarisation.	METTL3 mediates metabolic-immune crosstalk to promote an immunosuppressive microenvironment in endometriosis.	[391]
		Ectopic endometrial stromal cells (ESCs); patient tissues	MeRIP-seq; RNA immunoprecipitation (RIP); proliferation and invasion assays	m^6^A/IGF2BP2 (reader)—MEIS2/GATA6	Upregulated IGF2BP2 promotes m^6^A-dependent expression of MEIS2 and GATA6.	IGF2BP2 promotes ectopic implantation and tissue invasion in endometriosis.	[392]
	Adenomyosis	Endometrial stromal cells from patients with adenomyosis	Bioinformatics; qRT-PCR; immunohistochemistry; global m^6^A quantification	m^6^A/METTL3 (writer)—IGF1/DDT	METTL3 dysfunction lowers global m^6^A levels and is associated with increased IGF1 and DDT expression, enhancing epithelial proliferation and cell migration and promoting EMT-related programmes.	Reduced METTL3 and global m^6^A dysregulation may contribute to endometrial and myometrial dysfunction in adenomyosis via IGF1/DDT.	[393]

Abbreviations: ac^4^C, N4-acetylcytidine; acRIP-seq, acetylated RNA immunoprecipitation sequencing; A-to-I, adenosine-to-inosine; AUC, area under the curve; ChIP-seq, chromatin immunoprecipitation sequencing; circRNA, circular RNA; CTX, cyclophosphamide; DHT, dihydrotestosterone; ELISA, enzyme-linked immunosorbent assay; EMT, epithelial-to-mesenchymal transition; ESCs, endometrial stromal cells; GC, granulosa cells; GDM, gestational diabetes mellitus; GWAS, genome-wide association study; H3K27me3, histone H3 lysine 27 trimethylation; JAK/STAT, Janus kinase/signal transducer and activator of transcription; lncRNA, long non-coding RNA; m^1^A, N1-methyladenosine; m^5^C, 5-methylcytidine; m^6^A, N6-methyladenosine; m^7^G, N7-methylguanosine; MDSCs, myeloid-derived suppressor cells; MeRIP-seq, methylated RNA immunoprecipitation sequencing; MET, mesenchymal-to-epithelial transition; miRNA, microRNA; mRNA, messenger RNA; OXPHOS, oxidative phosphorylation; PAR-CLIP, photoactivatable ribonucleoside-enhanced crosslinking and immunoprecipitation; PCOS, polycystic ovary syndrome; PE, preeclampsia; piRNA, PIWI-interacting RNA; POI, primary ovarian insufficiency; Ψ, pseudouridine; qRT-PCR, quantitative reverse-transcription PCR; RIF, recurrent implantation failure; RIP, RNA immunoprecipitation; RNA-seq, RNA sequencing; ROC, receiver operating characteristic; ROS, reactive oxygen species; RSA, recurrent spontaneous abortion; TCGA, The Cancer Genome Atlas. Studies on types of RNA modifications and their molecular targets in reproduction action together show that RNA modifications coordinate post-transcriptional gene regulation during all phases of reproduction. Studies have revealed key m^6^A functions in the maternal-to-zygotic transition and early embryogenesis, as well as uniquely m^5^C and Ψ roles in folliculogenesis and placental angiogenesis. The processes through which different modifications communicate with one another are slowly being unravelled. The modification networks associated with RNA are not independent players. They operate as coordinated regulatory systems, working together in defiance of transcription and translation. This perspective not only addresses the issues associated with single-modification studies but also aids the development of models on comprehensive reproductive regulation.

## Data Availability

No new data were created or analysed in this study. Data sharing is not applicable to this article.
